# Maternal diabetes programs sexually dimorphic early-onset cardiovascular dysfunction in metabolically healthy offspring

**DOI:** 10.1016/j.xcrm.2025.102454

**Published:** 2025-11-11

**Authors:** Allan Zhao, Yuxia Wei, Eftychia Kontidou, Ali Mahdi, Paulo R. Jannig, Sara Torstensson, Hong Jiang, Alice Larsson, Aida Collado, Rawan Humoud, Jacob Grünler, David Ersgård, Buket Öztürk Esen, Xiaowei Zheng, Haojiang Lu, Eva Lindgren, Sanjiv Risal, Jian Zhao, Elisabet Stener-Victorin, Henrik Toft Sørensen, Lars Pedersen, John Pernow, Zhichao Zhou, Sofia Carlsson, Sergiu-Bogdan Catrina, Qiaolin Deng

**Affiliations:** 1 Department of Physiology and Pharmacology, Karolinska Institutet, Stockholm, Sweden; 2 Department of Molecular Biosciences, The Wenner-Gren Institute, Stockholm University, Stockholm, Sweden; 3 Institute of Environmental Medicine, Karolinska Institutet, Stockholm, Sweden; 4 Division of Cardiology, Department of Medicine, Solna, Karolinska Institutet, Stockholm, Sweden; 5 Department of Molecular Medicine and Surgery, Karolinska Institutet, Stockholm, Sweden; 6 Department of Clinical Epidemiology and Center for Population Medicine, Aarhus University, Aarhus, Denmark; 7 Department of Clinical Science, Intervention and Technology (CLINTEC), Karolinska Institutet, Stockholm, Sweden; 8 Division of Obstetrics and Gynecology, Karolinska University Hospital, Stockholm, Sweden; 9 Department of Cardiology, Karolinska University Hospital, Stockholm, Sweden; 10 Center for Diabetes, Academic Specialist Center, Stockholm, Sweden

**Keywords:** developmental programming, diabetes, offspring, cardiovascular disease, endothelial dysfunction, DOHaD, inheritance, metabolism, sex dimorphism

## Abstract

The incidence of cardiovascular disease (CVD) in young individuals is increasing. This alarming trend underscores the need to identify at-risk groups for preventive measures. Emerging evidence suggests that maternal diabetes increases the risk for metabolic diseases and early-onset CVDs in their offspring. However, the evidence is largely observational, limited by confounding factors, and lacks crucial mechanistic insight. Here, we combine experimental, epidemiological, and clinical approaches to disentangle the effects of maternal diabetes on offspring metabolism and endothelial function. In mice, we find that maternal hyperglycemia induces early-onset endothelial dysfunction specifically in male offspring, independent of metabolic disease. In humans, a case-control study and an epidemiological study confirm elevated risk of early-onset endothelial dysfunction and related CVDs in metabolically healthy sons of mothers with type 1 diabetes. Our findings identify an underrecognized risk group for early-onset CVDs and emphasize the importance of maternal conditions in shaping the cardiovascular health of future generations.

## Highlights

• Maternal diabetes programs early-onset endothelial dysfunction in sons but not daughters
• Endothelial dysfunction in sons develops independent of metabolic disease
• Increased vascular oxidative stress and arginase 1 are identified as a mechanistic driver
• These findings highlight an underrecognized risk group for future preventive strategies

## Graphical abstract

**Figure:**
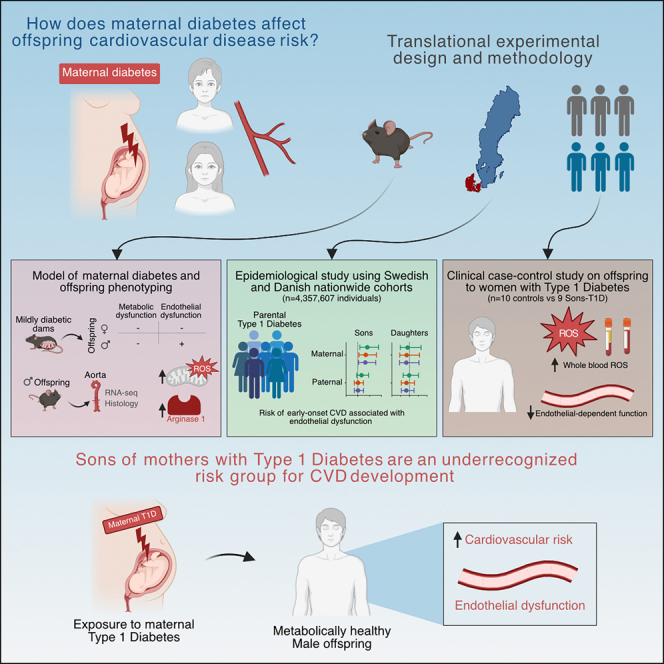

## Teaser

Zhao et al. translationally demonstrate how maternal diabetes induces early-onset endothelial dysfunction in sons, but not daughters, even when offspring are metabolically healthy. This challenges prior findings that metabolic dysfunction is the primary driver of cardiovascular disease in this population, guiding future clinical practice and risk management of these individuals.

## Introduction

The incidence of cardiovascular disease (CVD) in young individuals has steadily increased in recent decades, in contrast to the decreasing trend in older individuals,^1^ highlighting the need to further identify risk groups for targeted new preventive measures. CVD is associated with several well-known risk factors, including obesity, smoking, physical inactivity, and diabetes.^1^ Diabetes is a particularly important CVD risk factor and co-morbidity.^2^ Accumulating clinical and experimental evidence suggests that maternal diabetes and/or hyperglycemia increases cardiometabolic disease risk in offspring, through either germline transmission or developmental programming.^3^^,^^4^^,^^5^^,^^6^^,^^7^^,^^8^ Building on this knowledge, we have recently shown that in the context of maternal type 1 diabetes (T1D) with mild hyperglycemia, the uterine environment remains impaired and affected by hypoxia, thereby leading to fetal growth deviations and placental dysfunction.^9^ Interestingly, prenatal exposure to hypoxia has been associated with endothelial dysfunction in the offspring.^10^^,^^11^^,^^12^^,^^13^ Endothelial dysfunction develops as a result of imbalances in vasodilatory and/or vasoconstrictive properties,^14^ is an early hallmark of CVD development, and subsequently plays an important role in the development of CVDs such as hypertensive disorders, atherosclerosis, and ultimately myocardial infarction and stroke.^14^^,^^15^ Therefore, early-onset disturbances in endothelial function might be the underlying pathological factor reflecting the cause of the elevated CVD rates in offspring of mothers with T1D, defining this risk group.

Maternal diabetes significantly increases the risk of metabolic dysfunction in the offspring, which itself is a strong co-morbidity and risk factor for CVDs.^1^^,^^2^^,^^5^^,^^6^^,^^8^ Therefore, early development of metabolic dysfunction in offspring of mothers with diabetes has been speculated to be the mechanism driving the increase in CVDs in this population.^7^ In line with this hypothesis, it has been shown that the association between maternal diabetes and elevated blood pressure in young offspring was primarily mediated by offspring body mass index (BMI).^16^ However, these observational studies have not clarified whether CVD is a consequence of metabolic dysfunction or an independent event. Hence, it is possible that maternal diabetes predisposes their offspring to CVDs independent of metabolic dysfunction, thereby affecting a much larger population than previously postulated. This distinction has important implications for clinical practice and public health guidelines, particularly regarding whether all individuals exposed to maternal diabetes during pregnancy should be considered to be at elevated risk of developing early-onset CVD and targeted for preventive measures. Further studies are warranted to clarify this relationship and the underlying mechanisms in greater detail.

Previous experimental and clinical studies have indicated the presence of endothelial dysfunction in individuals exposed to maternal diabetes during gestation.^4^^,^^17^^,^^18^^,^^19^ However, those studies have also demonstrated clear concurrent metabolic dysfunction in the offspring, which might have preceded and subsequently contributed to the endothelial dysfunction as described above. Moreover, the rodent models used to delineate the mechanisms in the experimental studies have a clear limitation in that severe hyperglycemia and/or a single high dose of streptozotocin (STZ) during gestation can adversely affect overall maternal health and fetal development in a nonphysiological manner, thus potentially introducing additional confounding factors.^17^^,^^18^^,^^19^ Hence, further understanding of the interplay between metabolic and cardiovascular dysfunction in offspring of mothers with diabetes, by using more physiological animal models together with translational human evidence, is needed. Importantly, the characterization and investigation of endothelial function in offspring affected by maternal diabetes would pave the way to early risk-factor interventions and management to prevent future CVD development in this at-risk group.

Here, we used a mouse model of mild maternal hyperglycemia and demonstrate that their young and metabolically healthy male offspring develop early-onset endothelial dysfunction, whereas female offspring remained unaffected. Both *in vivo* and *ex vivo* functional studies pinpointed alterations in oxidative-stress- and arginase-1-related signaling as the underlying mechanisms. Importantly, we translated our findings through an epidemiological study, in which we used Danish and Swedish nationwide cohorts to demonstrate that sons, but not daughters, of mothers with T1D have an increased risk of developing early-onset CVDs related to endothelial dysfunction. Finally, we performed a clinical case-control study to provide further translational evidence of our findings, thus confirming the presence of early-onset endothelial dysfunction in male offspring of mothers with T1D.

## Results

### Characterization of mice with mild maternal T1D for mating

**Figure 1:**
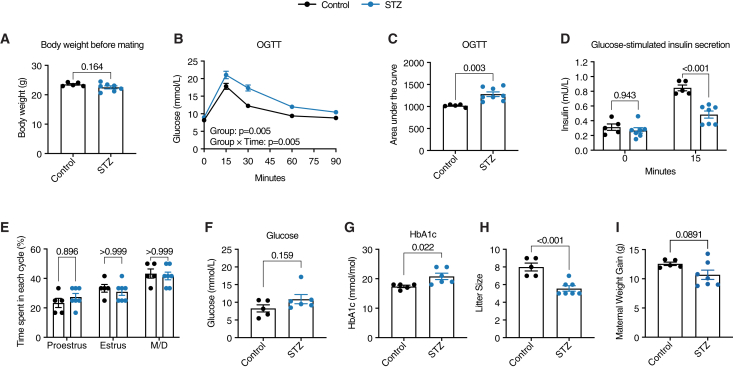
Characterization of maternal mice confirms mild T1D phenotype (A) Body weight of control (*n* = 5) and STZ (*n* = 7) mice before mating. (B) Blood glucose levels at different time points during an oral glucose tolerance test (OGTT). (C) Area under the curve (AUC) from the OGTT experiment in (B). (D) Plasma insulin levels at 0 and 15 min time points from OGTT experiment in (B). (E) Time spent in every phase of estrus cyclicity measurement. (F) Blood glucose levels at sacrifice. (G) Glycated hemoglobin (HbA1c) levels at sacrifice. (H) Litter size of liveborn pups. (I) Maternal weight gain during gestation for control (*n* = 5) and STZ (*n* = 7) mice. (A, C, F, G, H, and I) were analyzed using an unpaired Student’s t test, (B) was analyzed using a two-way ANOVA, and (D and E) were analyzed using a two-way ANOVA with Bonferroni’s post-hoc test. Data are represented as mean ± SEM.

The number of patients with T1D achieving better glycemic control and mitigated hyperglycemia during pregnancy is rapidly increasing, thus highlighting the importance of modeling and investigating effects in this group and future generations.^9^ Therefore, we utilized our optimized protocol of STZ injections to create diabetic female mice (STZ mice) with mild hyperglycemia. We have previously demonstrated how female mice subjected to this optimized protocol exhibit a milder state of hyperglycemia, which better reflects the clinical presentation of concurrent patients with diabetes. Importantly, these female mice lack common STZ-associated side effects and confounders, such as weight loss, off-target organ toxicity, and reproductive dysfunction, which otherwise could obscure intergenerational analyses.^9^ Use of these mice therefore allows us to strictly study the relationship between mild, clinically relevant maternal hyperglycemia and offspring outcomes. Similarly to our previous findings,^9^ we observed no body weight differences between the control and STZ mice at the time of mating (Figure 1A). The STZ mice had an impaired response to an oral glucose tolerance test (OGTT) with significantly lower glucose-stimulated insulin secretion (Figures 1B–1D) and did not differ in estrus cyclicity when compared to controls (Figure 1E).

**Figure 2:**
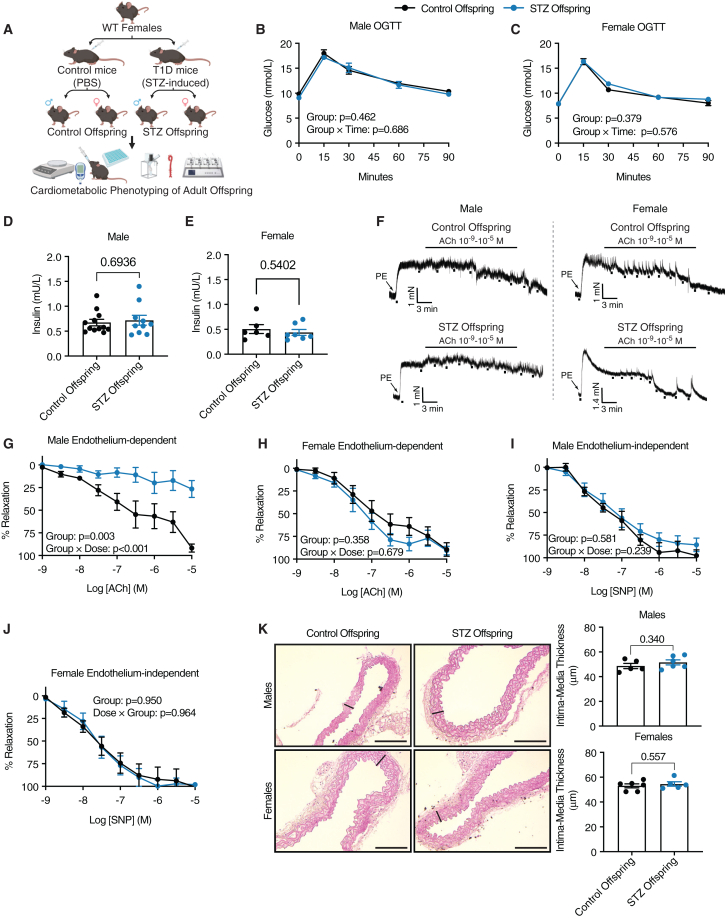
Exposure to maternal diabetes causes early-onset endothelial dysfunction in metabolically healthy male, but not female, offspring in mice (A) Schematic illustration of the experimental design. (B) Blood glucose levels at different timepoints during an OGTT for male offspring mice (*n* = 12, 5 litters and *n* = 10, 5 litters for control and STZ offspring, respectively). (C) Blood glucose levels at different time points during an OGTT for female offspring mice (*n* = 6, 3 litters and *n* = 7, 4 litters for control and STZ offspring, respectively). (D) Fasting insulin of male control (*n* = 12, 5 litters) and STZ offspring (*n* = 10, 5 litters). (E) Fasting insulin of female control (*n* = 6, 3 litters) and STZ offspring (*n* = 7, 4 litters). (F) Representative original tracings of endothelium-dependent relaxation (EDR) in mouse aortas analyzed using a wire myograph. (G) Endothelium-dependent relaxation (EDR) in mouse aortas from male offspring of control (*n* = 5, *n* = 3 litters) and STZ (*n* = 6, *n* = 3 litters) mice evoked with acetylcholine (ACh). (H) EDR in mouse aortas from female offspring of control (*n* = 6, *n* = 3 litters) and STZ (*n* = 6, *n* = 3 litters) mice evoked by ACh. (I) Endothelium-independent relaxation (EIR) in mouse aortas from male offspring of control (*n* = 3, *n* = 3 litters) and STZ (*n* = 4, *n* = 3 litters) mice evoked with sodium nitroprusside (SNP). (J) EIR in mouse aortas from female offspring of control (*n* = 4, *n* = 3 litters) and STZ (*n* = 4, *n* = 3 litters) mice evoked with SNP. (K) Representative H&E images of aortic segments and quantification of intima-media thickness in male offspring of control (*n* = 5, *n* = 3 litters) and STZ (*n* = 6, *n* = 3 litters) mice as well as in female offspring of control (*n* = 6, *n* = 3 litters) and STZ (*n* = 5, *n* = 3 litters) mice. Offspring mice were analyzed from at least three separate litters from different maternal mice. Data from (B, C, F, H, and I) were analyzed using a repeated measures two-way ANOVA. Data from (D, E, and G) were analyzed using an unpaired t test. The scale bar in the images denotes 100 μm. The images are marked with the area counted as intima-media thickness for each representative image. Data are represented as mean ± SEM.

Ten weeks after induction, we mated STZ and control female mice with healthy males to obtain STZ and control offspring (Figure 2A). At the end of the weaning period, the fasting glucose levels were similar between STZ and control mice, whereas the glycated hemoglobin (HbA1c) levels were slightly higher in the STZ mice, thus indicating that the mild increase in blood glucose previously observed in STZ mice^9^ was sustained throughout pregnancy and weaning (Figures 1F and 1G). Litters from STZ females were significantly smaller than those from control females, indicating a compromised intrauterine environment in agreement with previous observations when pregnancies were terminated at E18.5^9^ (Figure 1H), while the maternal weight gain was not significantly altered between the groups (Figure 1I).

### Offspring of STZ-induced mice are metabolically healthy at an early age

Next, we conducted a comprehensive metabolic characterization of the offspring of control mice (control offspring) and STZ mice (STZ offspring) at 4 months of age. This time point was chosen because it corresponds to early adulthood in humans and thus represents a suitable time window for studying early-onset cardiometabolic dysfunction.^20^ Interestingly, both male and female control and STZ offspring showed similar body weight gain up to 15 weeks of age (Figures S1A and S1B). We further assessed glucose tolerance in control and STZ offspring and observed no differences between groups for both males and females (Figures 2B, 2C, S1C, and S1E). In concordance with this, the plasma insulin levels at baseline and after glucose stimulation during the OGTT, as well as the fasting insulin levels, were similar between control and STZ offspring for both sexes (Figures 2D, 2E, S1D, and S1F).

We also performed indirect calorimetry to further investigate the whole-body energy homeostasis in control and STZ offspring. Again, neither male nor female STZ offspring differed from their respective control offspring in oxygen consumption, carbon dioxide production, energy expenditure, respiratory exchange ratio, cumulative food intake, or locomotor activity (Figures S2 and S3). Collectively, these results show how exposure to mild maternal T1D does not alter the metabolic function of either male or female offspring in young adulthood.

### Male, but not female, STZ offspring exhibit early-onset endothelial dysfunction

As STZ offspring of both sexes in our study were metabolically healthy at 4 months of age, this time point presented as a suitable time point to investigate their cardiovascular function to identify whether offspring endothelial dysfunction could exist even without concurrent metabolic dysfunction. Therefore, we next chose to evaluate the endothelium-dependent relaxation (EDR) and endothelium-independent relaxation (EIR) in aortas collected from control and STZ offspring at this time point by using a wire myograph to evaluate endothelial and smooth muscle cell function, respectively (Figures 2A and 2F).

Strikingly, we found that exposure to mild maternal hyperglycemia during pregnancy led to a severely impaired EDR in male offspring, with the aorta showing marked impaired relaxation even at high concentrations of the nitric-oxide-dependent vasodilator acetylcholine (ACh) (Figure 2G). In contrast, the EDR was normal in both female control and STZ offspring (Figure 2H), thereby highlighting a strong sexually dimorphic effect on endothelial function. The EIR, in contrast, was normal in both male and female STZ offspring, pinpointing the dysfunction in the endothelial layer, while smooth muscle function remains intact (Figures 2I and 2J).

Maternal diabetes is a risk factor for congenital malformations in offspring, which can also affect the vascular morphology.^21^^,^^22^ Therefore, to understand whether any structural or macroscopic alterations might have caused the endothelial dysfunction, we performed hematoxylin and erythrosine (H&E) staining of sectioned aortic rings. We observed no significant differences in the intima-media thickness of the aorta between control and STZ offspring for both sexes, suggesting that the impaired EDR has a molecular rather than structural origin (Figure 2K).

Because endothelial function naturally deteriorates with age,^23^ a predisposition to endothelial dysfunction might remain hidden in the early years of life and become exposed with aging. To ensure that EDR impairment did not develop at an older age in female STZ offspring, we analyzed aortas from female control and STZ offspring at a later time point (36 weeks of age). In agreement with our previous results, we observed no differences in the EDR or EIR between the two groups of female offspring at the later time point either, thus confirming the male-specific dysfunction observed (Figures S4A and S4B). Taken together, these results suggest that early-onset endothelial dysfunction develops specifically in metabolically healthy male offspring of diabetic females, whereas female offspring remain unaffected, even at older ages.

### Increases in vascular arginase 1 expression and oxidative stress drive endothelial dysfunction in STZ male offspring

**Figure 3:**
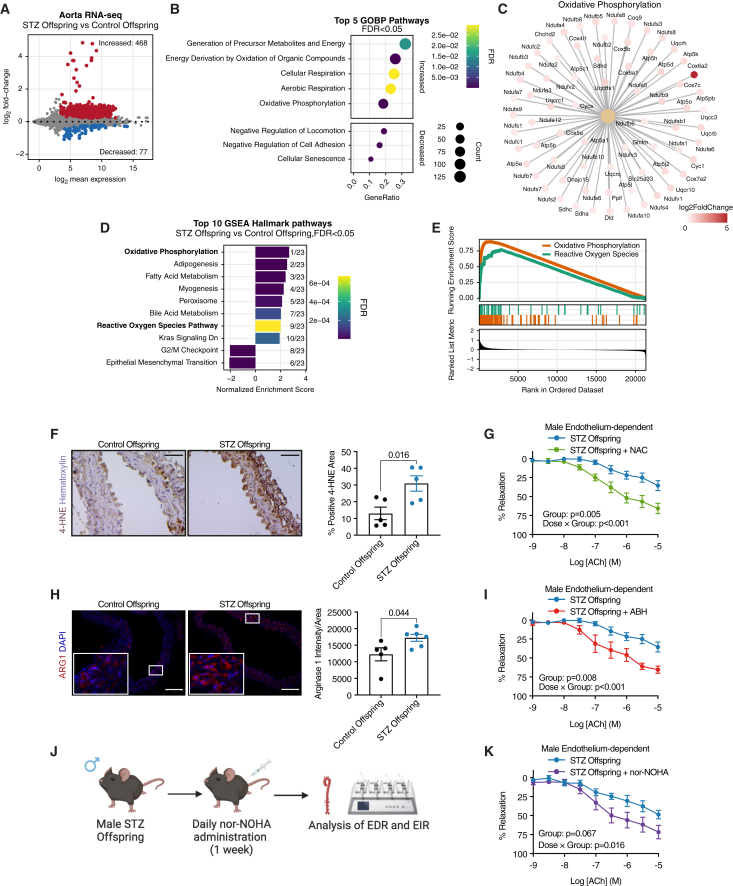
Molecular and functional analyses of aortas from male control and STZ offspring identifies alterations of oxidative stress and arginase 1 (A) MA plot depicting differentially expressed genes (DEGs; defined as adjusted *p* value < 0.05) obtained from bulk RNA-seq of aortas from male control (*n* = 5, *n* = 3 litters) and STZ (*n* = 6, *n* = 3 litters) offspring. (B) Bar plot depicting the top five enriched pathways from upregulated DEGs and three enriched pathways from downregulated DEGs in (A) analyzed with gene ontology (GO) overrepresentation test using GO biological process. (C) Network of significantly altered genes in the “oxidative phosphorylation” GO term. (D) Bar plot depicting the top 10 enriched pathways from a gene set enrichment analysis (GSEA) using Hallmark datasets comparing aorta from male control (*n* = 5, *n* = 3 litters) and STZ (*n* = 6, *n* = 3 litters) offspring. (E) Enrichment plots of the Hallmark oxidative phosphorylation and reactive oxygen species pathway gene sets generated through GSEA comparing aorta from male control (*n* = 5, *n* = 3 litters) and STZ (*n* = 6, *n* = 3 litters) offspring. (F) Representative images of immunohistochemical staining of 4-hydroxynonenal (4-HNE) in aortas; scale bar represents 50 μm; and quantification of % positive 4-HNE area in tissue sections of aortas from male control (*n* = 5, *n* = 3 litters) and STZ (*n* = 5, *n* = 3 litters) offspring. (G) EDR in aortas from male STZ offspring (*n* = 6, *n* = 3 litters) incubated with Krebs-Henseleit buffer (KHB, control) or the antioxidant N-acetylcysteine (NAC) for 1 h before measurement. (H) Representative images of fluorescent immunohistochemical staining of arginase 1 (Arg1) and DAPI in aortas; scale bars represent 100 μm; and quantification of Arg1 in tissue sections of aortas from male control (*n* = 5, *n* = 3 litters) and STZ (*n* = 6, *n* = 3 litters) offspring. (I) EDR in aortas from male STZ offspring (*n* = 6, *n* = 3 litters) incubated with KHB (control) or the arginase inhibitor 2(S)-amino-6-boronohexanoic acid (ABH) for 1 h before measurement. (J) Schematic illustration of experimental design for *in vivo* treatment of STZ male offspring using the arginase inhibitor *N*^ω^-hydroxy-nor-l-arginine (nor-NOHA). (K) EDR in aortas from male STZ offspring treated with PBS (*n* = 5, *n* = 3 litters) or nor-NOHA (*n* = 6, *n* = 3 litters) daily for 1 week. Offspring mice were analyzed from at least three separate litters from different maternal mice. (F and H) were analyzed using an unpaired t test. (G, I, and K) were analyzed using a repeated measures two-way ANOVA. Data are represented as mean ± SEM.

Subsequently, we sought to understand the mechanisms underlying the impaired EDR in male STZ offspring. Hence, we analyzed the transcriptome of aortas from male control and STZ offspring using bulk RNA sequencing (RNA-seq). Initial clustering using principal-component analysis (PCA) showed that the groups mainly differed along PC1 (Figure S5A). Differential expression analysis using DESeq2 identified 545 differentially expressed genes (DEGs), most (468/545) of which were increased in male STZ offspring (Figure 3A). Gene Ontology (GO) overrepresentation analysis of the upregulated DEGs revealed significant enrichment in terms related to oxidative phosphorylation and mitochondrial function, such as “cellular respiration,” suggesting increased mitochondrial activity in the aortas of male STZ offspring (Figure 3B). In contrast, the downregulated genes were enriched in only three terms: cell adhesion, senescence, and locomotion (Figure 3B). Moreover, most of the genes enriched in the mitochondria-associated pathways encoded for different subunits of the electron transport chain (Figure 3C). In line with this, rank-based gene set enrichment analysis (GSEA) using Hallmark gene sets^24^ identified “oxidative phosphorylation” as the top upregulated pathway (Figures 3D and 3E). Another pathway significantly upregulated in aortas from male STZ offspring was the reactive oxygen species (ROS) pathway, implying that the increased mitochondrial activity also leads to higher ROS accumulation in the aorta in male STZ offspring (Figures 3D and 3E).

Moreover, several of the upregulated DEGs, such as *Sod2*, *Aldoa*, *Taldo1*, *and Atox1* have previously been demonstrated to protect against excess oxidative stress^25^^,^^26^^,^^27^^,^^28^ (Figure S5B). The upregulation of these genes suggests activation of a compensatory response to the accumulation of ROS in STZ male offspring aortas. *Sod2* is of particular interest, as it encodes the most important SOD isoform for scavenging mitochondria-derived superoxide, indicating that the excessive ROS formation is mitochondrial in origin.^28^ Interestingly, increased ROS activity and oxidative stress can cause endothelial dysfunction.^29^ Therefore, the endothelial dysfunction observed in male STZ offspring could be due to excess oxidative stress caused by elevated mitochondrial activity. Moreover, individual mitochondrial complexes have different contributions to ROS generation, and complex I is a major source.^30^ Therefore, we performed overrepresentation analysis on our DEGs using MitoPathways gene sets to understand which complexes were predominantly affected in our data. Indeed, complex I was found to be the most affected mitochondrial subunit of all (Figure S5C). Our results hence indicate a preferential upregulation of genes-related mitochondrial complex I, which hypothetically could cause excessive ROS production, leading to development of endothelial dysfunction.

Although transcriptomic data provide clues about mechanisms, they do not constitute functional evidence. Therefore, to test our hypothesis, we next functionally investigated the relationship between elevated ROS and endothelial dysfunction in aortas obtained from control and STZ offspring males. We first analyzed the levels of 4-hydroxynonenal (4-HNE), a lipid peroxidation product and marker of oxidative stress, using immunohistochemistry. Indeed, the levels of 4-HNE were significantly higher in the aortas from STZ male offspring compared to controls (Figure 3F), confirming the increase in oxidative stress suggested by our transcriptomic results (Figures 3D, 3E, and S5B).

Next, we sought to investigate the functional implications of the elevated oxidative stress in male STZ offspring aortas. To address this, we applied the antioxidant N-acetylcysteine (NAC) to reduce oxidative stress in these aortas. We co-incubated aortic segments from male STZ offspring together with NAC for 1 h before assessing EDR using the wire myograph, while control vessels were incubated in Krebs buffer. Co-incubation of male STZ offspring aorta with NAC rescued the endothelial function, thus confirming the functional importance of ROS signaling (Figure 3G).

Sufficient L-arginine bioavailability is essential to maintain endothelial function, because this amino acid is used by the enzyme endothelial nitric oxide (NO) synthase (eNOS) to produce NO, which subsequently acts as an important anti-inflammatory and vasodilatory molecule.^29^^,^^31^ One mechanism underlying decreased L-arginine bioavailability involves increased levels of arginase 1 and 2, which convert L-arginine into L-ornithine, thereby decreasing eNOS activity and impairing EDR.^29^ Importantly, arginase activity can be increased by excessive oxidative stress, and increased arginase activity, in turn, causes eNOS uncoupling, resulting in increased production of superoxide.^29^ This creates a feedback loop, where higher levels of ROS impair the EDR even further.^29^ Interestingly, increased arginase expression and activity has been demonstrated to impair EDR in a variety of diseases, including type 2 diabetes (T2D),^32^ familial hypercholesterolemia,^33^ and COVID-19,^34^ highlighting its wide importance in maintaining vascular function. Therefore, we hypothesized that a pathological increase of arginase in STZ male offspring could further explain the impairment in EDR observed in this group.

We first investigated the expression of *Arg1* and *Arg2* in our transcriptomic data and observed no differences in *Arg1* between the groups, while *Arg2* expression was slightly decreased in aortas from STZ male offspring (Figures S6A and S6B). However, oxidative stress might influence arginase expression and activity at the post-translational level, as previously observed.^29^^,^^35^^,^^36^ Therefore, we next quantified the protein levels of both arginase 1 and 2 using fluorescent immunohistochemistry in aortas from both control and STZ male offspring. Interestingly, arginase 1 was significantly upregulated in aortas from STZ male offspring, suggesting its involvement in the impaired EDR (Figure 3H). In contrast, arginase 2 levels were similar between groups (Figures S6C and S6D).

To assess the functional relevance of the arginase 1 upregulation observed in aortas from male STZ offspring, we used the arginase inhibitor 2(S)-amino-6-boronohexanoic acid (ABH) to inhibit arginase 1, once again through co-incubation with aortic vessels for 1 h. Similarly to the co-incubation with NAC, ABH treatment significantly ameliorated the EDR impairment in aortas from male STZ offspring (Figure 3I). These results suggest that STZ male offspring have increased levels of vascular arginase and ROS, leading to significant impairment in EDR.

*Ex vivo* incubations are informative to dissect mechanisms but still lack the physiological and functional complexity that exists *in vivo.* Therefore, we next sought to investigate the therapeutic potential of arginase 1 inhibition *in vivo* in male STZ offspring. To do so, we administered the arginase inhibitor *N*^ω^-hydroxy-nor-l-arginine (nor-NOHA) intraperitoneally once daily for 1 week prior to sacrifice, followed by measurement of EDR (Figure 3J). Reassuringly, *in vivo* treatment with nor-NOHA significantly ameliorated the impaired EDR seen in male STZ offspring, mirroring the effects seen in *ex vivo* incubations (Figure 3K). Notably, the EIR remained normal in both groups (Figure S7A). Moreover, nor-NOHA showed no acute toxic effects, as evidenced by no differences in body weight or liver weight between the groups (Figures S7B and S7C). Taken together, these findings underscored vascular arginase as a promising therapeutic target to ameliorate EDR in sons of mothers with T1D.

### Nation-wide epidemiological cohort studies in Denmark and Sweden confirm increased risk of endothelial-dysfunction-related CVDs specifically in male offspring

As previously mentioned, a recent cohort study from Denmark has identified an elevated risk of early-onset CVDs among offspring of mothers affected by diabetes during pregnancy, including T1D.^7^ However, the investigated outcomes were based on all the CVD-associated codes from the International Classification of Diseases, 8^th^ and 10^th^ revisions (ICD-8 and ICD-10, respectively), which include a broad range of CVDs of various etiologies, such as infectious agents (e.g., endocarditis) and inflammation (e.g., pericarditis), many of which are largely unrelated to endothelial dysfunction.^7^ Moreover, the study did not adjust for offspring metabolic disorders such as T1D and T2D, although metabolic dysfunction might have affected the CVD risk in offspring, given that these are risk factors for many of the investigated outcomes.^7^

**Figure 4:**
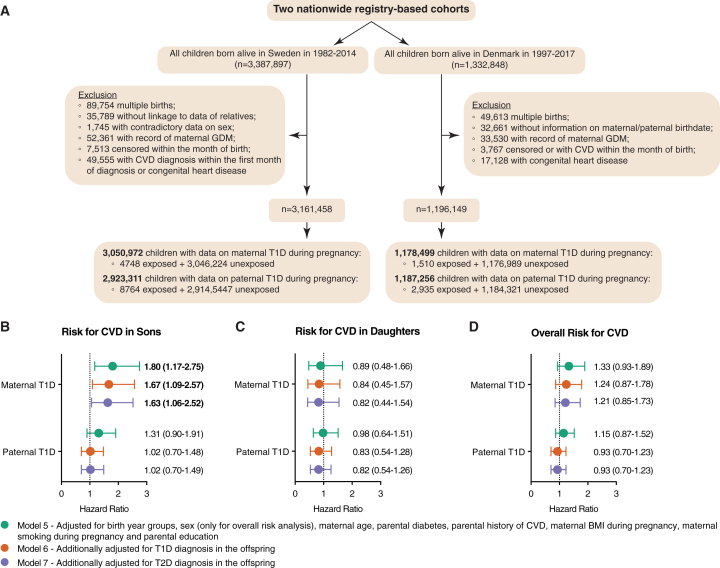
Maternal type 1 diabetes increases the risk of early-onset cardiovascular diseases associated with endothelial dysfunction specifically in male offspring (A) Flowchart depicting workflow for the two nationwide registry-based cohort studies included in this study. (B) Adjusted hazard ratios (HRs) and 95% confidence intervals (CIs) for the association between parental type 1 diabetes and risk for early-onset CVDs related to endothelial dysfunction in sons. (C) Adjusted HRs and 95% CIs for the association between parental type 1 diabetes and risk for early-onset CVDs related to endothelial dysfunction in daughters. (D) Adjusted HRs and 95% confidence intervals (CIs) for the association between parental type 1 diabetes and risk for early-onset cardiovascular diseases (CVDs) related to endothelial dysfunction in offspring. A total of *n* = 4,357,607 individuals were included in the two cohorts combined. The reference group was individuals unexposed to parental diabetes during pregnancy.

Hence, to investigate whether maternal T1D specifically affects endothelial-dysfunction-related CVDs in offspring independently of metabolic disorders, we conducted a new cohort study, combining nationwide data from Denmark and Sweden to increase the statistical precision. In our cohort, the exposure was maternal T1D, and the outcomes were restricted to ICD codes for only CVDs associated with endothelial dysfunction (full list of included diagnoses in Methods). To control for genetic effects, we also analyzed the effects of paternal T1D on offspring CVD risk. We included a total of 4,357,607 individuals (3,161,458 in Sweden and 1,196,149 in Denmark) born between 1982 and 2017, of whom 6,258 had mothers with T1D (4,748 in Sweden and 1,510 in Denmark) and 11,699 had fathers with T1D (8,764 in Sweden and 2,935 in Denmark) (Figure 4A). We identified 20,591 cases of CVD during follow-up for the maternal comparison (16,462 in Sweden and 4,129 in Denmark) and 19,448 cases of CVD for the paternal comparison (15,307 in Sweden and 4,141 in Denmark). The estimates were adjusted for gender, birth year groups, maternal age, paternal diabetes, parental history of CVD, maternal BMI, smoking status during pregnancy, and parental education (model 5). Moreover, we further adjusted for T1D (model 6) and T2D (model 7) diagnosis among offspring, to investigate the relation between maternal T1D and early-onset CVDs independently of metabolic disorders in the offspring. The cohort descriptions for the Swedish and Danish studies are shown in Tables S1 and S2.

Given the sexually dimorphic response observed in our animal model experiments (Figures 2G–2J), we wondered whether a similar pattern with one offspring gender being preferentially affected would emerge in our epidemiological data. Therefore, we first analyzed the effects of maternal T1D on offspring CVD risk according to offspring gender. Interestingly, we found a clearly elevated risk of development of early-onset CVDs related to endothelial dysfunction in male offspring of mothers with T1D (model 7, adjusted HR [aHR] 1.63, 95% confidence interval [CI] 1.06–2.52) but not in female offspring of mothers with T1D (model 7, aHR 0.82, 95% CI 0.44–1.54) (Figures 4B and 4C; Table 1). Moreover, paternal T1D was not associated with CVD risk in either male or female offspring (male model 7, aHR 1.02, 95% CI 0.70–1.49; female model 7, adjusted HR 0.82, 95% CI 0.54–1.26). These findings further imply that maternal-specific nongenetic factors, such as the uterine environment, play a more important role than genetic factors in the development of early-onset CVDs among offspring of mothers with T1D (Figures 4B and 4C; Table 1).

**Table 1:** Adjusted hazard ratios and 95% confidence intervals for the association between parental T1D and risk of early-onset cardiovascular diseases in the offspring

Exposures	No. of cases	Person years	Model 1^a^	Model 2^a^	Model 3^a^	Model 4^a^	Model 5^a^	Model 6^a^	Model 7^a^
**Sweden +Denmark**^b^									
**Overall**									
Maternal T1D during pregnancy	20,591	70,457,517	**1.45 (1.01–2.06)**	**1.45 (1.02–2.06)**	1.25 (0.76–2.05)	1.32 (0.93–1.87)	1.33 (0.93–1.89)	1.24 (0.87–1.78)	1.21 (0.85–1.73)
Paternal T1D during pregnancy	19,448	67,782,337	1.25 (0.95–1.66)	1.25 (0.95–1.66)	1.14 (0.86–1.51)	1.14 (0.86–1.52)	1.15 (0.87–1.52)	0.93 (0.7–1.23)	0.93 (0.7–1.23)

**Sons**									
Maternal T1D during pregnancy	10,226	36,186,721	**1.95 (1.27–2.99)**	**1.95 (1.27–2.99)**	1.63 (0.82–3.22)	**1.78 (1.16–2.74)**	**1.8 (1.17–2.75)**	**1.67 (1.09–2.57)**	**1.63 (1.06–2.25)**
Paternal T1D during pregnancy	9,703	34,818,423	1.43 (0.99–2.07)	1.43 (0.99–2.08)	1.31 (0.9–1.9)	1.31 (0.9–1.9)	1.31 (0.9–1.91)	1.02 (0.7–1.48)	1.02 (0.7–1.49)

**Daughters**									
Maternal T1D during pregnancy	10,365	34,270,795	0.99 (0.53–1.84)	0.99 (0.53–1.84)	0.89 (0.42–1.9)	0.9 (0.48–1.67)	0.89 (0.48–1.66)	0.84 (0.45–1.57)	0.82 (0.44–1.54)
Paternal T1D during pregnancy	9,745	32,963,914	1.06 (0.7–1.63)	1.06 (0.69–1.63)	0.97 (0.63–1.49)	0.98 (0.64–1.5)	0.98 (0.64–1.51)	0.83 (0.54–1.28)	0.82 (0.54–1.26)

**Sweden**									
**Overall**									
Maternal T1D during pregnancy	16,462	51,612,510	1.34 (0.88–2.04)	1.34 (0.88–2.04)	0.92 (0.43–1.99)	1.23 (0.81–1.87)	1.24 (0.81–1.88)	1.15 (0.75–1.75)	1.12 (0.73–1.70)
Paternal T1D during pregnancy	15,307	48,861,911	**1.37 (1.01–1.85)**	**1.37 (1.01–1.85)**	1.25 (0.92–1.69)	1.25 (0.92–1.70)	1.26 (0.93–1.70)	0.99 (0.73–1.34)	0.99 (0.73–1.33)

**Sons**									
Maternal T1D during pregnancy	8,222	26,523,913	**1.96 (1.20–3.21)**	**1.96 (1.20–3.20)**	**1.48 (0.50–4.42)**	**1.80 (1.10–2.95)**	**1.81 (1.11–2.96)**	**1.67 (1.02–2.74)**	1.63 (0.99–2.67)
Paternal T1D during pregnancy	7,687	25,116,129	**1.56 (1.05–2.33)**	**1.56 (1.04–2.32)**	1.42 (0.95–2.12)	1.42 (0.95–2.12)	1.42 (0.95–2.13)	1.07 (0.71–1.60)	1.07 (0.71–1.60)

**Daughters**									
Maternal T1D during pregnancy	8,240	25,088,596	0.73 (0.33–1.62)	0.73 (0.33–1.62)	0.46 (0.14–1.50)	0.67 (0.30–1.49)	0.67 (0.30–1.49)	0.63 (0.28–1.39)	0.61 (0.27–1.36)
Paternal T1D during pregnancy	7,620	23,745,782	1.17 (0.74–1.86)	1.17 (0.74–1.87)	1.07 (0.68–1.71)	1.08 (0.68–1.71)	1.08 (0.68–1.72)	0.90 (0.56–1.43)	0.89 (0.56–1.41)

**Denmark**									
**Overall**									
Maternal T1D during pregnancy	4,129	18,845,007	1.74 (0.9–3.34)	1.74 (0.91–3.35)	1.56 (0.81–3.00)	1.56 (0.81–3.00)	1.56 (0.81–3.01)	1.5 (0.78–2.89)	1.47 (0.76–2.82)
Paternal T1D during pregnancy	4,141	18,920,426	0.73 (0.35–1.53)	0.73 (0.35–1.53)	0.67 (0.32–1.41)	0.67 (0.32–1.41)	0.68 (0.33–1.43)	0.64 (0.31–1.34)	0.64 (0.31–1.35)

**Sons**									
Maternal T1D during pregnancy	2,004	9,662,808	1.91 (0.8–4.58)	1.91 (0.8–4.58)	1.73 (0.72–4.15)	1.72 (0.72–4.13)	1.75 (0.73–4.19)	1.67 (0.7–4.02)	1.65 (0.68–3.96)
Paternal T1D during pregnancy	2,016	9,702,294	0.87 (0.33–2.31)	0.87 (0.33–2.31)	0.81 (0.3–2.15)	0.81 (0.3–2.15)	0.83 (0.31–2.2)	0.77 (0.29–2.05)	0.78 (0.29–2.07)

**Daughters**									
Maternal T1D during pregnancy	2,125	9,182,199	1.58 (0.59–4.2)	1.58 (0.59–4.22)	1.4 (0.52–3.73)	1.4 (0.52–3.73)	1.37 (0.51–3.68)	1.32 (0.49–3.54)	1.28 (0.48–3.44)
Paternal T1D during pregnancy	2,125	9,218,132	0.61 (0.2–1.87)	0.61 (0.2–1.88)	0.55 (0.18–1.7)	0.55 (0.18–1.7)	0.56 (0.18–1.72)	0.52 (0.17–1.61)	0.52 (0.17–1.62)

Pooled and country-specific results from Sweden and Denmark. The reference group in all analyses was individuals unexposed to parental diabetes during pregnancy.

T1D, type 1 diabetes; CVD, cardiovascular disease.

Cox models with attained age as the time scale were used to HRs (95% CIs). Model 1 was adjusted for sex, birth year groups, and maternal age. Model 2 was additionally adjusted for paternal diabetes (no, T1D, or T2D) during the lifetime. Model 3 was additionally adjusted for parental CVD during the lifetime. Model 4 was further adjusted for maternal BMI during pregnancy. Model 5 was additionally adjusted for maternal smoking during pregnancy and parental education. Model 6 was additionally adjusted for T1D diagnosis in the offspring. Model 7 was additionally adjusted for T2D diagnosis in the offspring. The reference group in all analyses was individuals unexposed to parental diabetes during pregnancy.

a Hazard ratios (HRs) and 95% confidence intervals (95% CIs) are presented.

b The HRs (95% CIs) are pooled using fixed-effects meta-analysis.

We next sought to investigate how adjustment for offspring metabolic disorders affected the CVD risk in offspring exposed to parental T1D. When limiting the outcomes to CVDs related to endothelial dysfunction, we found that there was a slightly elevated risk of early-onset CVDs for individuals exposed to maternal T1D during pregnancy when combining the risks for both sons and daughters (model 7 aHR 1.21, 95% CI 0.85–1.73) but not for paternal T1D (model 7 aHR 0.93, 95% CI 0.70–1.23) (Figure 4B; Table 1). When not adjusting for offspring diabetes (model 5), there was indeed a slightly higher risk for early-onset CVDs for both the maternal T1D group (aHR 1.33, 95% CI 0.93–1.89) and the paternal T1D group (aHR 1.15, 95% CI 0.87–1.52) (Figure 4B; Table 1), confirming the importance of adjusting for offspring diabetes in these comparisons. Interestingly, albeit we still observed an elevated CVD risk for offspring exposed to maternal T1D before adjustment for offspring metabolic disorders, the risk estimates in these comparisons were imprecise and statistically non-significant, which contrasts with previous evidence.^7^ This could be explained by our further selection of endothelial-dysfunction-related ICD codes, rather than using all CVD-related ICD codes. Intriguingly, adjustment for offspring T1D diagnosis had a large attenuating effect in analysis of both maternal and paternal T1D in relation to offspring CVD (model 6), but almost no further attenuation was observed after adjustment for offspring T2D diagnosis (model 7), indicating that offspring T1D diagnosis contributes more than offspring T2D diagnosis to early-onset CVD development in T1D offspring (Figure 4D; Table 1). Notably, this finding was particularly evident for paternal T1D, thereby suggesting that early-onset CVD risk in the offspring of men with T1D is largely due to elevated risk of T1D among offspring. These observations highlight the importance of adjusting for metabolic disorders when investigating the associations between parental diseases and offspring CVD risk.

Overall, our epidemiological cohort study using Swedish and Danish data suggests that maternal T1D leads to a 63% elevated risk of early-onset CVDs related to endothelial dysfunction, specifically in male offspring, independent of offspring metabolic disorders.

### Case-control study confirms endothelial dysfunction among metabolically healthy sons of mothers with T1D

**Figure 5:**
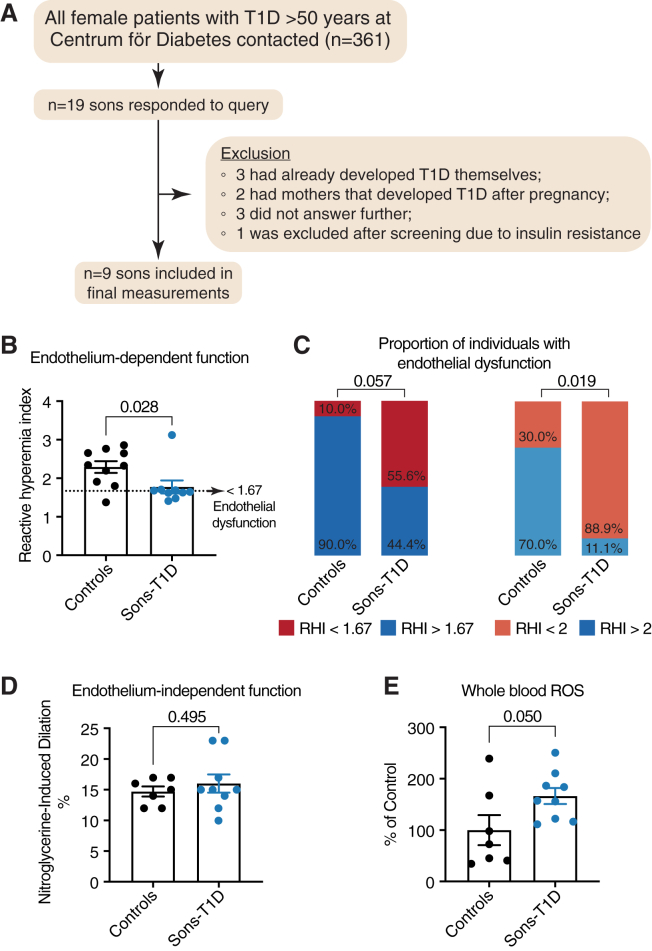
Case-control study of sons of mothers with type 1 diabetes (sons-T1D) and matched controls clinically verifies presence of early-onset endothelial dysfunction in metabolically healthy sons of women with type 1 diabetes (A) Flow chart depicting workflow, recruitment, and exclusion for the clinical study. (B) Analysis of endothelial function using reactive hyperemia index (RHI) in sons of mothers with T1D and matched controls. (C) Proportion of individuals with endothelial dysfunction (RHI < 1.67) in controls and sons of mothers with T1D and proportion of individuals with impaired endothelial function (RHI<2) in controls and sons of mothers with T1D. (D) Analysis of endothelium-independent vasodilation of the brachial artery after administration of sublingual nitroglycerine in controls and sons of mothers with T1D. (E) Analysis of whole blood reactive oxygen species (ROS) levels in controls and sons of mothers with T1D using electron paramagnetic resonance (EPR) spectroscopy. (B) was analyzed using a Mann-Whitney U test, (C) was analyzed using Fisher’s exact test, and (D and E) were analyzed using an unpaired Student’s t test. Data are represented as mean ± SEM.

To clinically verify the presence of early-onset endothelial dysfunction in male offspring to mothers with T1D, we performed a case-control study. Sons of mothers with T1D were recruited through a specialized diabetes clinic (Center for Diabetes) in Stockholm, Sweden. To ensure the translatability and coherence with our previous epidemiological and experimental findings, we focused on young adults (18–35 years of age). Moreover, we performed a comprehensive metabolic screening to exclude individuals with diabetes, insulin resistance, or other metabolic disturbances, to ensure a focus solely on metabolically healthy sons, in agreement with our previous experimental approach (Figure 5A; Table 2).

**Table 2:** Clinical characteristics of study subjects in case-control study

	Controls (*n* = 10)	Sons of mothers with T1D (*n* = 9)	*p* value
Age (years)	27.5 (27–30.5)	29 (22.5–30)	0.7971
BMI (kg/m^2^)	24.96 (24.27–25.73)	24.78 (22.76–28.48)	>0.9999
SBP (mmHg)	117.5 (111.8–130.3)	115 (108.5–120.5)	0.4586
DBP (mmHg)	69 (65.75–76)	71 (69–73.5)	0.6449
**Laboratory analyses**			
Hemoglobin (g/L)	136.5 (132.8–141.3)	144 (137.5–152.5)	0.0823
Thrombocytes (10^9^/L)	212 (197–231.8)	208 (186–256.5)	0.8881
Leukocytes (10^9^/L)	4.55 (4–5.825)	5 (4.4–5.7)	0.3646
Insulin (mIU/L)	5.7 (2.9–6.9)	5.4 (4.75–7.7)	0.757
Fasting glucose (mmol/L)	5.2 (4.875–5.45)	5.4 (5.1–5.65)	0.1914
HbA1c (mmol/mol)	34 (34–34)	32.5 (30–34)	0.1179
HOMA-IR	1.317 (0.612–1.717)	1.375 (1.08–1.852)	0.4698
Total cholesterol (mmol/L)	4.05 (3.975–4.35)	4.5 (3.85–5.15)	0.5091
LDL (mmol/L)	2.3 (2.2–2.575)	2.8 (2.25–3.2)	0.2486
HDL (mmol/L)	1.45 (1.175–1.7)	1.2 (1.1–1.65)	0.4342
Triglycerides (mmol/L)	0.655 (0.5275–0.895)	0.7 (0.66–1.095)	0.1887
Creatinine (umol/L)	87.5 (78.75–93.5)	81 (72–100)	0.6166
hsCRP (mg/L)	0.415 (0.2–0.5875)	0.36 (0.285–0.705)	0.8416

BMI, body mass index; SBP, systolic blood pressure; DBP, diastolic blood pressure; HbA1c, glycated hemoglobin; HOMA-IR, homeostatic model assessment for insulin resistance; LDL, low-density lipoprotein; HDL, high-density lipoprotein; hsCRP, high-sensitivity C-reactive protein. *p* values are stated for the comparison between controls and sons of mothers with T1D. Variables presented as median and interquartile ranges. Variables analyzed with Mann-Whitney U test.

In total, we identified 19 sons of mothers with T1D, nine of whom met our inclusion criteria (Figure 5A). For a control group, we recruited 10 age-matched sons whose mothers were not affected by diabetes during pregnancy. No significant differences in age, BMI, or blood pressure were observed between the groups (Table 2). Moreover, no significant differences were observed in any of the laboratory parameters analyzed, including routine bloodwork as well as metabolic parameters such as HbA1c, fasting blood glucose, fasting insulin levels, lipid levels, and HOMA-IR (Table 2). Hence, the groups were clinically and metabolically well matched, thereby allowing for the analysis of vascular function without potential confounding factors.

Thereafter, we assessed the peripheral endothelial function in both groups by measuring the reactive hyperemia index (RHI) using peripheral arterial tonometry and analyzed endothelium-independent vasodilation after sublingual nitroglycerine administration. RHI is a measure of endothelial function based on the principle of reactive hyperemia, and lower RHI is associated with atherosclerotic risk factors and future CVD events.^37^ In line with our experimental findings, we observed a significant impairment of endothelial function in healthy sons of mothers with T1D as indicated by a lower RHI compared to matched controls (healthy sons of mothers without disorders during pregnancy) (Figure 5B). Moreover, among sons of mothers with T1D, a larger proportion displayed impaired endothelial function (RHI <2.0), with a trend also suggesting higher proportion of endothelial dysfunction (RHI <1.67) compared to controls^37^^,^^38^ (Figure 5C). Importantly, the vasodilation after sublingual nitroglycerine administration did not differ between the healthy sons of mothers with T1D and controls, highlighting that endothelial dysfunction is present in sons of mothers with T1D, whereas the endothelium-independent response and vascular smooth muscle function remained intact, similar to the observations in our mouse studies (Figures 5D, 2G, and 2H).

Finally, to verify a key mechanism underlying the endothelial dysfunction observed in our mouse model (Figures 3F and 3G), we analyzed whether healthy sons of mothers with T1D had higher circulating markers of oxidative stress. Indeed, whole blood ROS measurement with electron paramagnetic resonance (EPR) spectroscopy revealed a 66% increase in ROS levels in sons of mothers with T1D when compared to controls, indicating that the endothelial dysfunction in this population is associated with elevated oxidative stress (Figure 5E).

## Discussion

The interplay between diabetes, endothelial dysfunction, and CVDs is well known. Recent evidence have suggested that maternal diabetes can program offspring to have an increased risk of developing CVDs, but the mechanisms have remained largely unknown.^7^ In this study, we applied our mouse model of mild hyperglycemia^9^ to investigate the effects of maternal T1D on offspring endothelial function. This is an important strength of this work, as the properties of our model enabled us to study direct effects of maternal hyperglycemia in the absence of the conditions that are frequently present in other models and might potentially influence the offspring CVD risk. Because endothelial dysfunction is often an early-onset manifestation of later-onset CVD,^14^ we hypothesized that this might be detected even in metabolically healthy STZ offspring. Unexpectedly, we observed a sexually dimorphic effect, wherein only male STZ offspring had impaired endothelial function—a finding attributed to dysregulation of arginase 1 and oxidative stress. Importantly, we confirm that targeting arginase *in vivo* is a promising therapeutic approach to ameliorate endothelial dysfunction in male STZ offspring. Moreover, we validated our findings in the human medical context by performing an epidemiological cohort study using Danish and Swedish nationwide registries, as well as a case-control study investigating healthy sons of mothers with T1D and matched healthy sons not affected by maternal diabetes during pregnancy. Our findings collectively demonstrate that sons of mothers with T1D have elevated risk of developing endothelial dysfunction and related CVDs, highlighting this population as a previously underrecognized group at risk of CVD development, which might have major implications on clinical practice.

Our findings have several important clinical implications. Endothelial dysfunction is a well-established precursor of common CVDs such as atherosclerosis and hypertension, which are associated with high morbidity and mortality.^14^^,^^15^ Because we used a mouse model reflecting appropriate glycemic management, our findings suggest that the improved glycemic control currently observed in patients before and during gestation might not be sufficient to prevent harmful effects in male offspring. This is consistent with recent epidemiological data suggesting that the risk for pregnancy-related complications remains elevated in patients with T1D and appropriate glycemic management.^39^^,^^40^ These findings suggest potentially persistent effects on subsequent generations even under improved maternal metabolic conditions in T1D.

Another clinically relevant finding is that the endothelial dysfunction in sons developed regardless of any concurrent metabolic dysfunction. Since we observed this association in our epidemiological and clinical studies even after adjusting for offspring metabolic conditions, these findings imply that all male offspring, and not only those with metabolic illness, might have elevated risk of CVDs. Consequently, all sons born to mothers with T1D, regardless of the level of maternal glycemic control, should be considered a high-risk group for CVD. Pregestational diabetes is estimated to affect 1%–2% of all pregnancies, with a majority of it being T1D.^41^ The size of this risk group is therefore substantial and is expected to continue to increase with the growing incidence and prevalence of T1D.^42^^,^^43^ This warrants further attention for prevention and patient education.

A major strength of this work is our nationwide epidemiological cohort study using Swedish and Danish data, which suggests that maternal T1D is associated with a 63% elevated risk of early-onset CVDs in metabolically healthy male offspring. Importantly, long-term follow-up data are often a limiting factor in studies examining intergenerational effects, such as the effects of parental diseases on offspring outcomes. Herein, long-term follow-up was made possible by the unique, reliable, and high-quality nationwide registries containing vast medical information available in Nordic countries such as Sweden and Denmark.

Strikingly, in our clinical study, 89% of the sons of mothers with T1D displayed impaired endothelial function (RHI <2), and 56% of the sons of mothers with T1D displayed endothelial dysfunction (RHI <1.67). These proportions are unexpectedly high, despite the low number of participants, and highlight a clinically important vascular dysfunction in these individuals even before the onset of metabolic dysfunction. Interestingly, the proportions of endothelial dysfunction observed in our current study are similar to, or even greater than, those reported in conditions clearly associated with CVD risk and endothelial dysfunction, such as post-acute COVID-19 syndrome (PACS),^38^ rheumatoid arthritis,^44^ and even manifest diabetes.^45^ Therefore, sons of mothers with T1D might have similar levels and risks of endothelial damage and dysfunction to people with other, well-known endothelial-dysfunction-related disorders.

Our findings might also extend to individuals exposed to maternal T2D or gestational diabetes (GD), because these conditions similarly expose the developing fetus to hyperglycemia during gestation. Furthermore, our previous findings imply that the gestational exposure to hyperglycemia is the pathological driver.^9^ Indeed, a previous clinical study also identified endothelial dysfunction in offspring of patients with T2D.^4^ However, the participants also displayed clear metabolic and inflammatory alterations, which might have preceded and contributed to the development of endothelial dysfunction.^4^ Further research is needed to determine whether offspring to women with GD or T2D are also at risk of endothelial dysfunction even when metabolically healthy or if this association pertains to only offspring of mothers with T1D.

Our study identified excess arginase 1 and oxidative stress as underlying mechanisms to the EDR impairment in male offspring; however, additional pathways are likely to contribute. For instance, a study using a rat model of GD, established by a single high-dose STZ injection at the beginning of gestation, identified that endoplasmic reticulum (ER)-stress-related pathways induced endothelial dysfunction and hypertension in offspring.^18^ However, it is noteworthy that this was also accompanied by clear metabolic dysfunction.^18^ Moreover, the study focused on maternal animals with more severe hyperglycemia, which is less clinically relevant given the recent advancements in glucose-lowering therapeutics. The one-time high-dose STZ injection administered during gestation may also affect the developing fetus and other maternal organs,^46^ potentially confounding the observed effects on offspring phenotypes. Therefore, additional studies using models of milder hyperglycemia, without such confounding factors, are needed to further validate the role of ER stress in this context.

Our findings show that alterations in arginase 1 levels might serve as attractive therapeutic targets for ameliorating impaired EDR in male offspring of mothers with T1D. Another time point that might be of interest when it comes to preventive measures is the prenatal period, where correcting the maternal milieu might prevent developmental programming. The placental hypoxia observed by us may be linked to increased levels of placental oxidative stress, given that both conditions are frequently interconnected in the context of diabetes.^47^^,^^48^ Indeed, previous work has shown that NAC treatment during pregnancy can decrease the rate of congenital malformations in mice affected by severe hyperglycemia.^49^ Similarly, increased oxidative stress has been implicated in the pathogenesis of CVDs in maternal obesity.^50^ Investigating the role of oxidative stress induced by mild maternal diabetes during pregnancy is of importance to evaluate its potential as a target for preventive intervention.

The sexually dimorphic effects in outcomes observed in offspring are of high interest. Notably, in humans, males generally develop CVDs earlier than females.^51^^,^^52^ Although the molecular mechanisms underlying this disparity are not entirely understood, sex hormones are thought to play a role.^48^ For instance, estrogen increases the levels of the antioxidant enzyme glutathione peroxidase, which helps ameliorate oxidative stress.^51^ As our findings suggest increased oxidative stress as one of the key factors causing endothelial dysfunction specifically in the male offspring, the higher antioxidant capacity in females might explain why their EDR is maintained. Interestingly, it has recently been shown that many cardiometabolic risk factors change around the age of menopause, further highlighting the potential protective importance of estrogen in females.^53^ These connections are highly interesting and require further elucidation in future studies with longer follow-up periods for offspring.

We have previously demonstrated that embryos from STZ dams are growth restricted when compared to controls, pinpointing a persisting pathological uterine environment in mild hyperglycemia.^9^ Interestingly, growth restriction induced by other insults, such as hypoxia, protein restriction, or general nutrient restriction during gestation, has also been demonstrated to affect offspring endothelial function negatively.^12^^,^^54^^,^^55^^,^^56^ This highlights how various insults that affect fetal development may have a similar phenotype, both in the early and late stages. However, many of these previous reports did not observe the sex-dimorphic effects identified here, which then hypothetically could be more related to hyperglycemia *per se*.

In conclusion, our findings collectively demonstrate that maternal T1D predisposes male, but not female, offspring to early-onset endothelial dysfunction. This finding was functionally shown to depend on elevated arginase 1 levels and excessive oxidative stress. In line with this, we confirm that arginase inhibition is a promising strategy for treatment of endothelial dysfunction in this group using *in vivo* approaches. We translate our findings by identifying an identical sexually dimorphic association in clinical registries from Danish and Swedish nationwide cohorts, as well as in a case-control study comparing sons of mothers with T1D and matched controls. Importantly, we found that the impairment of male offspring EDR is independent of any concurrent offspring metabolic disease, thus highlighting all male offspring of mothers with T1D as a previously underrecognized risk group for CVD development. Altogether, our results underscore the importance of maternal conditions in shaping CVD risk and emphasize the need for interventions and patient risk management to mitigate the developmentally programmed CVD risk in future generations.

### Limitations of the study

Several limitations in our study should be highlighted. Our mouse model did not include exogenous insulin supplementation, which is typically seen in women with mild T1D. Instead, the model maintains mild hyperglycemia through remaining endogenous insulin production.^9^ However, the glycemic levels and dynamics, which are important pathological factors in humans, were mirrored in our mouse model. Moreover, the mechanism behind the hyperglycemia in our mouse model is slight hypoinsulinemia, which is inherent to any STZ-induced diabetic mouse model. This may itself affect metabolic processes other than carbohydrate metabolism, such as fat metabolism. However, it is important to note that the baseline insulin levels at a non-challenged state were comparable between control and STZ mice^9^ (Figure 1D). Future studies are nonetheless necessary to assess potential alterations in fat metabolism, such as plasma triglyceride levels, as we cannot completely exclude the possibility that these factors may confound the offspring phenotype. Furthermore, our study is based on a single type of diabetes model, namely the optimized STZ-induced model, and did not include another model of T1D, either genetic or induced by a structurally unrelated pharmacological compound. Although our previous work^9^ has shown that common STZ-related side effects and confounders are absent in our model, it is still of high relevance to confirm our findings in independent models of diabetes. In the current work, we were also not able to delineate the mechanisms underlying the observed difference in endothelial function between sons and daughters of mothers with T1D, which is of large interest and should be investigated further in follow-up studies. Moreover, the low power of our epidemiological study resulted in broad CIs across all analyses. Nevertheless, we still observed a significant association between maternal T1D and an elevated risk of endothelial-dysfunction-related CVDs among male offspring. Finally, although our clinical case-control study serves as a proof of concept, it included relatively few participants, and further research with larger cohorts is needed to validate and extend these findings. We can also not exclude possible unknown confounders such as undiagnosed coronary disease in the participants. However, this is uncommon in the current age population. Finally, we did not rescue the endothelial dysfunction in our human case-control study, as any pharmacological intervention would require further ethical approval and hence lies beyond the scope of our current work, but it remains an important next step to explore the potential for future therapeutic intervention.

## Resource availability

### Lead contact

Further information and requests for resources and reagents should be directed to and will be fulfilled by the lead contact, Qiaolin Deng (qiaolin.deng@ki.se).

### Materials availability

This study did not generate new unique reagents.

### Data and code availability

• The raw sequencing data for mouse aorta RNA sequencing reported in this paper have been deposited under the Bioproject and SRA accession no. PRJNA1193260.
• Codes that support our results and figures will be available at GitHub upon publication at www.github.com/Denglab-KI as well as on Zenodo, https://doi.org/10.5281/zenodo.17091392.
• Any additional information required to reanalyze the data reported in this work is available from the lead contact upon request.

## Acknowledgments

We thank the Histocore, Metabolic Phenotyping Center at the Strategic Research Program in Diabetes, and Comparative Medicine-B at Karolinska Institutet. We also thank all the group members of the Deng laboratory for constructive feedback and discussions during meetings. This work was mainly supported by the grants to Q.D., including 10.13039/501100004359Swedish Research Council (no. 2018-02557 and 2020-00253), 10.13039/501100004973Barndiabetesfonden, and 10.13039/501100008550Diabetesfonden. Q.D. is also funded as a Wallenberg Academy Fellow in Medicine and through faculty funding at 10.13039/501100004047Karolinska Institutet. Other funding includes the 10.13039/501100004359Swedish Research Council (2020-01372 and 2024-02534 to J.P., 2022-00811 to S.C., 2023-02508 to Z.Z., and 2022-00550 to E.S.-V.); the EFSD/Novo Nordisk Foundation Future Leader Award (to Z.Z.); the 10.13039/501100003793Swedish Heart Lung Foundation (20220264, 20230386, and 20240072 to Z.Z., 20220210 to J.P., and 20241002 to S.-B.C.); Stockholm County Council ALF (088725 to A.M., 20190031 and FOUI-972326 to J.P., and 20220674 to S.-B.C.); 10.13039/501100008550Diabetesfonden and the Distinguished Investigator Grant – Endocrinology and Metabolism from Novo Nordisk Foundation (to E.S.-V.); the Foundation for Geriatric Diseases (to A.C.); Lars Hierta’s Memory Foundation (to A.C.); Tore Nilson’s Foundation for Medical Research (to A.C.); Kung Gustav V:s och Drottning Victorias Frimurarestiftelse (to S.-B.C.); Berth von Kantzows Foundation (to X.Z. and S.-B.C.); Erik Mattsons Foundation (to X.Z.); Rolf Luft Foundation (to X.Z.); and 10.13039/501100004047Karolinska Institutet grants (to Q.D., X.Z., S.-B.C., E.S.-V., A.C., and Z.Z.). A.Z. is supported by KID funding given to Q.D. and Forskar-AT-funding from 10.13039/501100004047Karolinska Institutet. H.J. is supported by a grant from the Chinese Scholarship Council. E.K. and R.H. are supported by KID funding given to Z.Z.

## Author contributions

Q.D. and A.Z. conceived and designed the overall study. Q.D. was the overall supervisor of this work. A.Z. performed the animal experiments with help from P.R.J., S.T., A.L., J.G., H.L., and S.R. A.Z. and E.K. performed the vessel phenotyping experiments with help from A.M., A.C., and Z.Z. A.Z. and A.L. prepared tissue sections and performed stainings. A.Z. and J.Z. performed microscopy. A.Z. and A.L. analyzed images. A.Z. prepared libraries for RNA sequencing. A.Z., P.R.J., and H.J. analyzed RNA sequencing data. A.Z., Y.W., S.C., and Q.D. conceived and designed the epidemiological study, to which B.Ö.E., H.T.S., and L.P. provided input. Y.W. and B.Ö.E. analyzed epidemiological data. A.Z., A.M., J.P., S.-B.C., and Q.D. conceived and designed the clinical case-control study with input from D.E. A.Z., D.E., and P.R.J. recruited patients and performed analyses. R.H. analyzed blood ROS levels. X.Z., E.S.-V., H.T.S., L.P., J.P., Z.Z., S.C., S.-B.C., and Q.D. provided conceptual and methodological suggestions and feedback. P.R.J. and A.Z. assembled figures, to which all authors provided input. A.Z., P.R.J., and Q.D. wrote the manuscript, to which all other authors provided input.

## Declaration of interests

The authors declare no competing interests.

## STAR★Methods

### Key resources table

**Table:** 

REAGENT or RESOURCE	SOURCE	IDENTIFIER
**Antibodies**		

Rabbit polyclonal anti-arginase 1	Sigma Aldrich	HPA003595; RRID: AB_1078190
Rabbit polyclonal anti-arginase 2	Sigma Aldrich	HPA000663; RRID: AB_1078192
Mouse monoclonal anti-4-hydroxynonenal	R&D Systems	MAB3249; RRID: AB_10556156
Donkey anti-rabbit Alexa Fluor 647	ThermoFisher	A-31573; RRID: AB_2536183

**Biological samples**		

Human whole blood	Study participants	This study
Mouse aorta	Mouse	This study
Mouse Plasma	Mouse	This study

**Chemicals, peptides, and recombinant proteins**		

Sudan Black-B	Sigma-Aldrich	199664
DAPI	ThermoFisher	D1306
CMH (1-hydroxy-3-methoxycarbonyl-2,2,5,5-tetramethylpyrrolidine hydrochloride)	Noxygen Science Transfer & Diagnostics GmbH	NOX-02.1-50mg
EPR-grade Krebs HEPES buffer	Noxygen Science Transfer & Diagnostics GmbH	NOX-7.6.1-500mL
Deferoxamine	Noxygen Science Transfer & Diagnostics GmbH	NOX-09.1-100mg
DETC (diethyldithiocarbamate)	Noxygen Science Transfer & Diagnostics GmbH	NOX-10.1-1g
Streptozotocin (STZ)	Sigma	S0130
ProLong Gold Antifade Mountant	Invitrogen	P36930
Maxima H Minus Reverse Transcriptase	ThermoFisher	EP0752
Exonuclease I	NEB	M0293S
KAPA HiFi RM	Roche	KR0370
SPRI-Select Beads	Beckman Coulter	B23317
Sera-Mag Speed Beads	GE HealthCare	65152105050250
N-acetylcysteine	Sigma-Aldrich	A7250
2(S)-amino-6-borohexanoic acid (ABH)	Sigma-Aldrich	SML1466
Sodium nitroprusside	Sigma-Aldrich	71778
Acetylcholine	Sigma- Aldrich	A5751
Potassium Chloride (KCl)	VWR	26764.298

**Critical commercial assays**		

Serum insulin (ELISA kit)	Crystal Chem	90080
Qubit 1x dsDNA HS Assay Kit	Invitrogen	Q33231
Bioanalyzer High Sensitivity DNA kit	Agilent	5067–4626
Reliaprep RNA extraction kit	Promega	Z6011
EnVision+ Dual Link System-HRP	Agilent Technologies/Dako	K4065
DCA HbA1c Reagent Kit for DCA Vantage	Siemens Healthcare	10004366
RNA 6000 Nano Kit	Agilent Technologies	5067–1511
NEBNext Ultra II FS DNA Library Prep kit	New England Biolabs	E6177

**Deposited data**		

Bulk-RNAseq	This study	BioProject: PRJNA1193260

**Experimental models: Organisms/strains**		

C57BL/6JRj mice	Janvier Labs	RRID:MGI:5751862

**Oligonucleotides**		

Template Switching Oligo (TSO) (E5V7NEXT)	Janjic et al.^58^	N/A
Barcoded oligodT (E3V7NEXT)	Janjic et al.^58^	N/A
Preamp Primer (SINGV6)	Janjic et al.^58^	N/A
Prime-seq Adapter Duplex	Janjic et al.^58^	N/A
i7 Index Primers (Nextera)	Janjic et al.^58^	N/A
I5 Index Primers (TruSeq)	Janjic et al.^58^	N/A

**Software and algorithms**		

R	https://www.r-project.org/	version 4.4.0
Stata statistical software 14.0	Stata Corps	RRID: SCR_012763
GraphPad Prism 10	GraphPad	RRID:SCR_002798
zUMIs pipeline^59^	https://github.com/sdparekh/zUMIs	version 2.9.7
DESeq2^60^	https://github.com/thelovelab/DESeq2	version 1.44.0
ImageJ (Fiji)	https://imagej.net/ij/	
clusterProfiler^61^	https://github.com/YuLab-SMU/clusterProfiler	version 4.8.2
MSigDB^62^	https://www.gsea-msigdb.org/gsea/msigdb	version 2023.1
Seurat^63^	https://github.com/satijalab/seurat	version 4.4.0
STAR^64^	https://github.com/alexdobin/STAR	version 2.7.10a
Cutadapt	https://cutadapt.readthedocs.io/en/stable/	version 4.1
FastQC	https://github.com/s-andrews/FastQC	version 0.11.9
RSubread^65^	https://github.com/ShiLab-Bioinformatics/subread	version 2.12.0
Code from this manuscript	https://doi.org/10.5281/zenodo.17091392	N/A

### Experimental model and study participant details

#### Ethical approval

All procedures and studies presented herein were conducted according to the guidelines of the Declaration of Helsinki and approved by appropriate authorities. The registry-based study in Sweden was approved by the Swedish Ethical Review Authority (ethical permit approval number 2023-06373-01). According to Danish law, analyses of registry data do not require approval from an ethical committee. However, the study received approval from Aarhus University on behalf of the Danish Data Protection Agency (record number 2016-051-000001, serial number 2679). All data for the Danish registry-based study were analyzed on a secure remote server provided by Statistics Denmark.

The clinical case-control study was approved by the Regional Ethics Committee of Stockholm (ethical permit approval number 2018/583-31 with amendment 2023-05792-02). Informed consent was obtained from all study participants.

All animal experiments were approved by the Stockholm Ethical Committee for Animal Research (17538-2020 with amendments 18959-2021 and 8639-2022), in accordance with the legal requirements of the European Community (SJVFS 2017:40) and the 2010/63/EU directive of the European Parliament on the protection of animals used for scientific purposes. Animal care and procedures were performed in accordance with guidelines specified by the European Council Directive and controlled by Comparative Medicine Biomedicum, Karolinska Institutet, Stockholm, Sweden.

#### Epidemiological cohort study – Registry linkage

To identify the Swedish cohort we used the Medical Birth Register (1973–2014) which collects information from the antenatal, obstetric, and neonatal records of 98% of all births in Sweden.^66^ The registry data were linked to the Prescribed Drug Register (2005–2015),^67^ the National Patient Register (including inpatient (1964–2015),^68^ the National Diabetes Register (1996–2020)^69^ and the Longitudinal Integration Database for health insurance and labor market studies (1970–2014).^70^ We used the Multi-Generation Register to link children to their parents.^71^ The cohort included all singletons born in Sweden from 1982 to 2014 (*n* = 3,387,897). We excluded multiple births (*n* = 89,754), children who died, were censored (*n* = 7513), developed any outcome within the first month of birth or had congenital heart disease (ICD-8 code 746 and 747; ICD-9 code 745, 746, 747A, 747B, 747C, 747D and 747E; or ICD-10 codes Q20-Q26^22^ diagnosed at birth or later (*n* = 49,555), individuals failing to be linked to the Multi-Generation Register for relatives’ information (*n* = 35,789), individuals with contradictory gender information (*n* = 1,745) or missing information in Medical Birth Register and Multi-Generation Register, individuals with missing data on exposures, or individuals with any record of maternal gestational diabetes (ICD-10 code O24.4) during any pregnancy (*n* = 52,361). For the final cohort, the follow-up years were 1982–2015, meaning the age of the subjects at follow-up ranged between 1 and 33 years of age. 51% of these individuals were men, and 49% were females (Table S1).

The Danish cohort was identified through the Danish Medical Birth Registry which includes more than 99% of births in Denmark since 1973.^72^ Additional information on parental variables was obtained through linkage with the following national registries: The Danish Civil Registration System,^73^ the Danish National Patient Registry,^74^ the Danish National Prescription Registry and the registries at Statistics Denmark on individual-level sociodemographic factors.^75^ The individual registries have been described in a previous study.^76^ The cohort included all singleton births in Denmark between 1997 and 2017 (*n* = 1,332,848). We excluded multiple births (*n* = 49,613), individuals without information on maternal/paternal birthdate (*n* = 32,661), individuals with any record of maternal gestational diabetes (*n* = 33,530), individuals who were censored or developed a CVD within the month of birth (*n* = 3,767), and individuals affected by congenital heart disease (*n* = 17,128). For the final cohort, the follow-up years were 1997–2018, meaning the age of the subjects at follow-up ranged between 1 and 21 years of age. 51% of these individuals were men, and 49% were females (Table S2). A flowchart describing the workflows and exact numbers for both cohorts can be found in Figure 4A.

#### Diabetes assessment

Information on diabetes in the parents and offspring was obtained from National Diabetes Patient, and Prescribed Drug Registers in the Swedish cohort, and the Danish National Patient and Prescription Registries in the Danish cohort. T1D was defined as a diagnosis of T1D in the Diabetes Register (Sweden only) or Patient Registers (ICD-10 code E10) or a record of diabetes at age ≤30 years, without any record of oral glucose-lowering drug use (ATC code A10B) or conflicting types of diabetes recorded. T2D was defined as a diagnosis of T2D in the Diabetes Register (Sweden only) or Patient Registers (ICD 10 code E10), or a diagnosis of diabetes (ICD-9 and 8 code 250) together with records of oral glucose-lowering drug. People with conflicting types of diabetes recorded (E10 and E11) were classified as having T2D if they had at least one record of oral glucose lowering drugs. Information on gestational diabetes was defined as ICD-9 code 648A, ICD-10 code O24.4 in the Patient Registers or Medical Birth Registers. Date of diabetes diagnosis was defined as the first date of diabetes record or the first date of glucose-lowering drug prescription.

An individual was considered exposed to maternal T1D during pregnancy if their mother was diagnosed with T1D before conception. The date of conception was estimated at 16 days (the median of 11–21 days) after the first day of the mother’s last menstrual period. The first day of the last menstrual period was calculated as the date of childbirth minus the gestational age. For cases without gestational age data, the date of conception was approximated as 9 months before the birth month. Their unexposed counterparts were individuals without any maternal diabetes (including gestational diabetes) before birth. An individual was defined as being exposed to paternal T1D during the mother’s pregnancy if the father was diagnosed with T1D before conception. Their unexposed counterparts were offspring who had no paternal diabetes before birth.

#### Cardiovascular outcome assessments

For the outcomes in the epidemiological analyses, we selected CVDs related to endothelial dysfunction through the National Patient Register (Sweden) and the Danish National Patient Registry. Early-onset CVD (before age 40) was identified using ICD codes for hypertensive diseases (ICD-10: I10-I15; ICD-9: 401–405; ICD-8: 400–404), ischemic heart disease (ICD-10: I20-I25; ICD-9: 410–414; ICD-8:410-414); pulmonary heart disease and diseases of pulmonary circulation (ICD-10: I26-I27; ICD-9: 415–416; ICD-8: 426); heart failure (ICD-10: I50; ICD-9: 428); cerebrovascular diseases (ICD-10: I60-I69; ICD-9: 430–438; ICD-8: 430–438); atherosclerosis (ICD-10: I70; ICD-9: 440; ICD-8: 440); arterial embolism and thrombosis (ICD-10: I74; ICD-9: 444; ICD-8: 444); venous thrombosis (ICD-10: I80-I82; ICD-9: 451–453; ICD-8: 450–453).

#### Covariates

Potential covariates included gender, birth year, birth weight, gestational age, birth order, mode of delivery, maternal age, maternal BMI during early pregnancy, maternal smoking during pregnancy, parental education, maternal lifetime CVD diagnosis, paternal lifetime CVD diagnosis, paternal diabetes (T1D, T2D or no; only when the exposure is maternal T1D during pregnancy), maternal diabetes (T1D, T2D or no; only when the exposure is paternal T1D during pregnancy), and offspring T1D and T2D.

#### Animals

Nine-week-old wild-type female C57BL/6JRj mice were obtained from Janvier Labs. All mice were maintained under a 12h light/dark cycle in a temperature-controlled room with *ad libitum* access to water and food. At 10 weeks of age, diabetes was induced as previously described using Streptozotocin (Sigma-Aldrich).^9^ After at least 10 weeks of disease exposure, mice were used for mating to generate offspring. All mice were fed the SDS 801722 CRM (P) diet from Special Diets Services, composing of 79.2% cereals, 17.6% vegetal proteins, 3.0% vitamins & minerals and <1% amino acids, with 68.9% of energy derived from carbohydrates, 22.03% from proteins, and 9.08% from fats.

#### Clinical study population

Nineteen male individuals with a mother with T1D (ages 18–45) were contacted through Centrum för Diabetes, Academic Specialist Center, Stockholm, Sweden. After excluding individuals who had developed diabetes themselves, displayed clear insulin resistance (HOMA-IR > 2.9), had a mother who developed diabetes after pregnancy and individuals who did not want to follow the investigation, *n* = 9 sons of women with T1D were included in the study (ages 18–33). Age-matched metabolically healthy subjects without any exposure to maternal diabetes of any kind were recruited as controls (*n* = 10) (ages 20–33). All participants were informed about the purpose and possible risks of the study and gave their informed consent.

### Method details

#### Measurement of estrus cyclicity

For assessment of reproductive function, the estrus cyclicity was characterized in STZ and control female mice as previously described.^9^

#### Oral glucose tolerance tests

Glucose metabolism and uptake were measured through an oral glucose tolerance test after a 5-h fast for both maternal and 15-week-old offspring mice. D-glucose was administered orally by gavage (2 g/kg) and blood glucose was measured using a glucometer (FreeStyle Precision) at 0, 15, 30, 60 and 90 min 30 μL of blood was collected at 0 and 15 min for plasma insulin measurement.

#### Plasma insulin measurement

Plasma insulin of blood samples obtained from the OGTT of both maternal and offspring mice was analyzed using an ELISA kit (Crystal Chem) according to the manufacturer’s instructions. A total of 5uL of plasma was used for analysis.

#### Measurement of glycosylated hemoglobin levels

Glycosylated hemoglobin levels (HbA1c) were measured using a DCA HbA1c Reagent Kit (Siemens Healthcare) on a DCA Vantage Analyzer (Siemens Healthcare) according to the manufacturer’s instructions.

#### Indirect calorimetry in metabolic cages

Metabolic parameters including oxygen consumption, carbon dioxide production, energy expenditure, respiratory exchange ratio, food intake and spontaneous locomotor activity were assessed using metabolic cages (TSE PhenoMaster, TSE Systems) in animals 15 weeks of age. Animals were kept in the metabolic cages single-housed for four consecutive days, with 48-h readings being used for analysis after a 24-h adaptation period. Analysis was subsequently done in CalR with the remove outliers feature activated.^77^

#### Nor-NOHA *in vivo* treatment experiment

For investigation of how the arginase inhibitor *N*^ω^-hydroxy-nor-*l*-arginine (nor-NOHA) affects endothelial function, 15 week-old male STZ offspring mice were injected with 40mg/kg nor-NOHA daily for 1 week. Controls were injected with PBS. Nor-NOHA (Bachem, Bubendorf, Switzerland) was dissolved in double-distilled water, sterile filtrated through a Millipore filter and tested for bacterial toxins and sterility. Weight was registered daily, and mice were sacrificed after 1 week of treatment for vascular measurements.

#### Wire myography

Aortas from 4-month-old offspring mice were collected after sacrifice in Krebs-Henseleit (KH) buffer, before being washed and cleaned by removing fat and connective tissues, before being cut into smaller (2 mm) segments. Thereafter, aortic segments were mounted on a wire myograph (Danish Myo Technology A/S) in separate 6 mL organ baths containing KH buffer. Assessment of endothelium-dependent relaxation (EDR) was performed through the application of acetylcholine in increasing concentrations (10^−9^ to 10^−5^ M) in incremental steps to preconstricted aortic segments. Preconstriction was performed with phenylephrine. Assessment of endothelium-independent relaxation (EIR) was performed using addition of incremental levels of sodium nitroprusside (10^.−9^ to 10^−5^ M).

For further characterization of the mechanisms underlying the impaired EDR in male STZ offspring aortas, aortic segments were co-incubated with antioxidant N-acetylcysteine (NAC, 10 μM) or the arginase inhibitor 2(S)-amino-6-borohexanoic acid (ABH, 100 μM) for 1 h before measurement of EDR. Control segments were incubated for the same period of time in KH buffer.

#### Tissue sectioning

Freshly collected aortic segments were fixed in 4% paraformaldehyde for 24 h at room temperature (RT) before dehydration and embedding. Paraffin-embedded and formalin-fixed aortic segments were then cut in 5 μm sections using a waterfall microtome (HM360, Microm). The sections were stretched in a 37°C–41°C water bath, transferred to a SuperFrost Microscope Slide (ThermoFisher), dried at 40°C for 1–2 h and at RT overnight.

#### Hematoxylin and erythrosine staining

For morphological analysis of aortas using hematoxylin and erythrosine staining, tissue sections were deparaffinized in xylene before rehydration using graded ethanol. Tissues were then stained with Mayer’s hematoxylin for 6 min and differentiation with acid ethanol (70% EtOH, 1% HCl). Nuclear blueing was then done using Scott’s tap water for 1 min before counterstaining using 0.3% erythrosine for 6 min. Thereafter, samples were dehydrated in graded ethanol, cleared in xylene and mounted using pertex.

#### Fluorescent immunohistochemistry

Fluorescent immunohistochemistry was performed on aortic segments. Briefly, deparaffinization and rehydration was performed, before antigen retrieval using pH6 Citrate buffer (Sigma Aldrich) in a pressure cooker. Samples were blocked with 10% donkey serum for 30 min at RT and then incubated with a rabbit polyclonal anti-arginase 1 antibody (1:100 dilution, catalog number HPA003595, Atlas Prestige Antibody, Sigma Aldrich) or a rabbit polyclonal anti-arginase 2 antibody (1:50 dilution, catalog number HPA000663, Atlas Prestige Antibody, Sigma-Aldrich) overnight at 4°C. Thereafter, sections were washed before incubation for 1h at RT using a fluorochrome-conjugated donkey anti-rabbit Alexa Fluor 647 secondary antibody (ThermoFisher) diluted 1:500. Subsequently, autofluorescence was quenched using 0.1% Sudan Black-B solution (Sigma) for 10 min, before counterstaining with DAPI (1:5000) for 3 min. Mounting was performed using ProLong Gold Antifade mounting media (ThermoFisher), before drying at RT for 24 h and thereafter at 4°C until imaging.

Imaging of the stained slides was performed using the LSM700 microscope (Zeiss) using z stack imaging at 20× magnification.

#### Immunohistochemistry

For immunohistochemistry, aortic sections were deparaffinized and rehydrated before antigen retrival using pH6 Citrate buffer (Sigma Aldrich) and a pressure cooker. Peroxidase inactivation was performed followed by blocking using goat serum (Abcam) for 30 min at RT, before incubation with a mouse monoclonal anti-4-hydroxynonenal (4-HNE, 1:100 dilution, catalog number MAB3249, R&D Systems, Minneapolis, MN) overnight at 4°C. Specific labeling was then detected using a labeled horseradish peroxidase (HRP) polymer conjugate as a secondary antibody as a part of the EnVision+ Dual Link System-HRP (Dako, Agilent Technologies). Isotype IgG (mouse IgG_2B_, Abcam) was used as a negative control to confirm antibody specificity. Samples were then developed using a solution containing 3, 3′-diaminobenzidine (Dako) before counterstaining with Mayer’s Modified Hematoxylin (Abcam) and mounted using mounting medium (Abcam). Images were captured using a DM3000 Digital microscope (Leica), and fields from each aortic section were captured.

#### Image analysis

All image analysis was blinded.

The intima-media-thickness was calculated using Fiji for each segment. Each image was measured at a minimum of 4 individual spots, and at least 3 images per aorta were analyzed.

For the quantification of 4-HNE in aortic segments, the area stained for 4-HNE and the total area of the aortic segment was manually calculated using Fiji. Adventitial layers were not included for quantification. The area positive for 4-HNE was then divided with the total area of the aortic segment.

For the analysis of the level of Arginase 1 and 2 staining in the aortic rings, the total intensity of staining was quantified in Fiji and divided to the total area of the aortic segment being analyzed.

#### RNA isolation

Snap-frozen aortas were lyzed in Trizol (Sigma Aldrich) using a RETCH MM 400 Mixer Mill (Fisher Scientific) for 4 × 2 minutes at 25 Hz. The samples were then incubated at RT for 15 min, before addition of 140 μL of chloroform. Thereafter, the samples were incubated for 3 min at RT, and subsequently centrifuged at 4°C for 15 min 350 μL of the aqueous layer was retrieved and mixed 1:1 with isopropanol, and RNA purification was performed using the ReliaPrep RNA MiniPrep System (Promega) according to the manufacturer’s instructions. The RNA was eluted in 15 μL nuclease-free water and quantified with a Nanodrop 1000 spectrophotometer (ThermoFisher) and a Qubit RNA quantification kit (ThermoFisher). Analysis of RNA integrity was performed using the Agilent Bioanalyzer RNA 6000 Pico Kit (Agilent Technologies), and only samples with an RNA integrity number (RIN) of 7 or above were used for library construction.

#### Library preparation for bulk RNA sequencing

Libraries for bulk RNA sequencing were prepared using prime-seq as previously described.^58^ Reverse transcription was performed using 40 ng RNA per sample before digestion of residual primers using Exonuclease I. Then, cDNA pre-amplification and second-strand synthesis were performed, followed by a cDNA quality and quantity control using the Qubit dsDNA HS 1X Assay Kit (ThermoFisher) and the Bioanalyzer High Sensitity DNA kit (Agilent). Once the cDNA was confirmed to be of good quality, the library was constructed using the NEBNext Ultra II FS Kit (New England Biolabs) according to protocol. Library quantity and quality was once again analyzed using Qubit and Bioanalyzer, before sequencing on an Illumina NovaSeq 6000 at an average depth of 10 million reads per sample.

#### Determination of endothelial function in humans

Individuals who agreed to participate in the study were scheduled for a study visit at the Cardiology Research Department at Karolinska University Hospital, Stockholm, Sweden. Participants were asked to refrain from eating and caffeinated beverages at least 6 h before the visit. Endothelial function was analyzed using the EndoPAT device (Itamar-Medical, Caesarea, Israel) with fully automated post-occlusion reactive hyperemia. The measurement of endothelial function is based on the principle of pulse amplitude tonometry (PAT) following reactive hyperemia induced by a 5-min occlusion of the Dig II fingertip using supra-arterial pressure. To obtain the reactive hyperemia index (RHI), the PAT signal 1–2 min after reactive hyperemia is divided by the baseline PAT before any cuff inflation. This acts as a surrogate for endothelial function. The contralateral fingertip (Dig II) served as a control. Endothelial dysfunction was defined as RHI <1.67, and impaired endothelial function was defined as RHI <2.0, in line with previous evidence.^37^^,^^38^^,^^78^

#### Determination of endothelium-independent vasodilation in humans

Endothelium-independent vasodilation was determined in the subjects by measureing the quota of the increase in brachial artery diameter after application of sublingual nitroglycerine (0.4 mg, PharmaPol), as previously described.^57^

#### EPR spectroscopy for ROS detection in whole blood

ROS formation in whole blood was determined using electron paramagnetic resonance (EPR) spectroscopy as previously described.^48^^,^^79^ Briefly, 26 μL of freshly collected blood was incubated with 1-hydroxy-3-methoxycorbonyl-2,2,5,5-tetramethylpyrrolidine (CMH, 200 μM), deferoxamine (25μM) and diethyldithiocarbamate (5 μM, Noxygen Science Transfer & Diagnostics GmbH, Elzach, Germany) for 30 min at 37°C before snap-freezing in liquid nitrogen and storage at −80°C. Measurements of samples were performed using a table-top EPR spectrometer (Noxygen Science Transfer & Diagnostics GmbH, Elzach, Germany) with the following settings: microwave frequency 9.752 GHz, modulation frequency 86 kHz, modulation amplitude 1.44 G, sweep width 100.00 G, microwave power 2.03 mW, number of scans 10. ROS levels was then calculated as % of control levels based on the average of the control samples.

### Quantification and statistical analysis

#### Statistical analysis of epidemiological data

The follow-up time was calculated from birth to the occurrence of the outcomes, emigration, death, or the date of turning age 40 years or December 31^st^, 2015 (Swedish data)/December 31^st^, 2022 (Danish data), whichever came first.

Cox models were used to compute the hazard ratios of CVD with 95% CI in relation to maternal T1D during pregnancy and paternal T1D during the pregnancy, with cluster-robust standard errors to correct for the correlation among children born by the same mother. Model 1 was adjusted for gender, birth year, and maternal age (model 1). Model 2 was further adjusted for paternal diabetes (no, T1D (overruling), or T2D) during the lifetime when the exposure is maternal T1D during pregnancy, and adjusted for maternal diabetes (no, T1D (overruling), or T2D) during the lifetime when the exposure is paternal T1D during the mothers’ pregnancy. Model 3 was additionally adjusted for parental CVD during the lifetime. Model 4 was further adjusted for maternal BMI during pregnancy. Model 5 was additionally adjusted for maternal smoking during pregnancy, and parental education. Model 6 additionally adjusted for T1D in the offspring. Model 7 was further adjusted for offspring T2D. Participants with missing values on continuous covariates were assigned the median values, with a binary variable to indicate whether the values are imputed or not. Participants with missing values on categorical variables were assigned as a separate group, and participants with missing values on continuous variables were assigned the population median values, with an additional variable to indicate whether the values were imputed or not.

In addition, we performed the analysis of parental T1D and offspring CVD risk separately in sons and daughters separately to explore potential gender differences.

We pooled the HRs from the Swedish and Danish cohorts using fixed-effects meta-analysis. The pooled results are referred to in the text, whilst cohort-specific results can be found in Table 1.

#### Sequencing data processing and analysis

Raw sequencing data was controlled using FastQC (version 0.11.9). Trimming of poly(A) tails and filtering was performed using Cutadapt (version 4.1) and the zUMIs pipeline (version 2.9.7).^63^ STAR^64^ (version 2.7.10a) was used for mapping of filtered data to the mouse genome, and the reads were counted using RSubread (version 2.12.0).^65^

Data analysis was performed in R, version 4.4.0. Quality of samples were checked using Seurat (version 4.4.0),^63^ and a sample with <3 x 10^6^ counts was excluded from further analysis due to it being less deeply sequenced compared to other samples. Differential gene expression analysis was performed using DESeq2 version 1.44.0.^60^ Mitochondrial and ribosomal genes were excluded from further analysis. Moreover, genes expressed in less than 5 samples (n of the smallest group) were removed from further analysis. Differentially expressed genes were defined by an adjusted *p*-value <0.05. Fold changes were shrunk using lfcShrink() and ashr.^80^ Then whole gene lists were ranked on log2 fold change and used for gene set enrichment analysis (GSEA) using the clusterProfiler package (v4.8.2)^61^ and curated hallmark gene sets from the Molecular Signatures Database (MSigDB, v.2023.1).^62^ Gene ontology (GO) overrepresentation analysis was performed on upregulated genes using clusterProfiler. MitoPathways overrepresentation analysis was performed on upregulated genes using the enricher() function in clusterProfiler using data from MitoCarta 3.0.^81^

#### Statistical analysis of other data

Statistical analysis of results apart from transcriptomic and epidemiological findings were performed in GraphPad Prism (version 9). Normality of data was evaluated using the Kolmogorov-Smirnov test. Differences between two groups were assessed using the Mann-Whitney U test (if data was not normally distributed), or a two-sided Student’s t test (if data was normally distributed). two-way ANOVA was used to assess the differences between groups when time was a second factor (OGTT, insulin measurements, myograph experiments). ANCOVA was used for analysis of data obtained from indirect calorimetry experiments with total mass as a covariate. Fisher’s exact test was used for analysis of differences in proportion of individuals. *p* < 0.05 was considered statistically significant.

#### Graphics

The graphical abstract and illustrations in Figures 2 and 3 were created using BioRender.com.

## Supplemental information

Document S1. Figures S1–S7 and Tables S1, and S2

Document S2. Article plus supplemental information
