# Supplementary material for: Maternal diabetes programs sexually dimorphic early-onset cardiovascular dysfunction in metabolically healthy offspring
Source: Cell Rep Med. 2025 Nov 11;6(11):102454. doi: 10.1016/j.xcrm.2025.102454 (PMC12711690; doi:10.1016/j.xcrm.2025.102454)
Supplement: Document S1. Figures S1–S7 and Tables S1, and S2 [file mmc1.pdf]

Cell Reports Medicine, Volume *6*

Supplemental information

Maternal diabetes programs sexually dimorphic early-onset cardiovascular dysfunction in metabolically healthy offspring

Allan Zhao, Yuxia Wei, Eftychia Kontidou, Ali Mahdi, Paulo R. Jannig, Sara Torstensson, Hong Jiang, Alice Larsson, Aida Collado, Rawan Humoud, Jacob Grünler, David Ersgård, Buket Öztürk Esen, Xiaowei Zheng, Haojiang Lu, Eva Lindgren, Sanjiv Risal, Jian Zhao, Elisabet Stener-Victorin, Henrik Toft Sørensen, Lars Pedersen, John Pernow, Zhichao Zhou, Sofia Carlsson, Sergiu-Bogdan Catrina, and Qiaolin Deng

Supplemental Materials

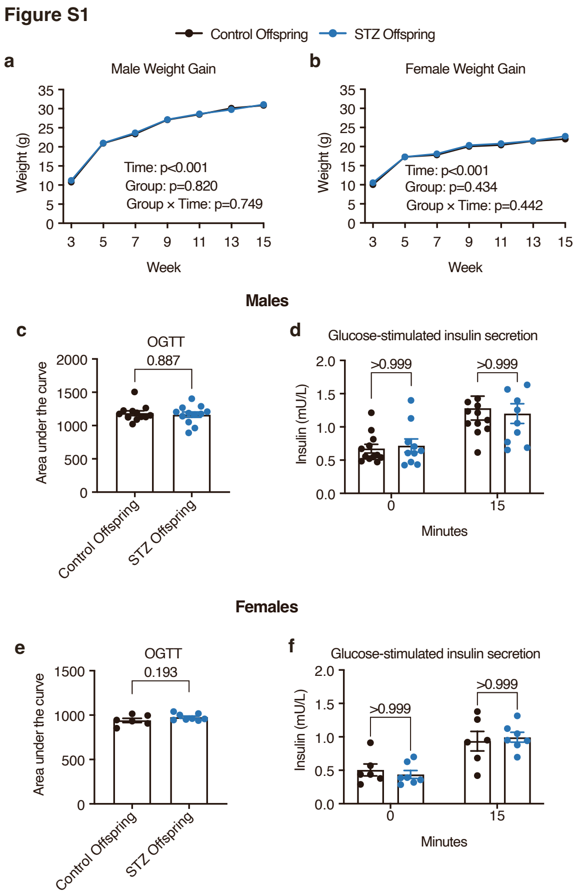

Figure S1. Metabolic analysis of male and female offspring to control and STZ mice finds no clear metabolic alterations. Related to Figure 2. a, Weight gain curve for male control (n=18, n=5 litters) and STZ offspring (n=19, n=7 litters) up to 15 weeks of age. **b,** Weight gain for female control (n=22, n=5 litters) and STZ offspring (n=21, n=7 litters) up to 15 weeks of age. **c,** AUC from the OGTT experiment in males (n=12, 5 litters and n=10, 5 litters for control and STZ offspring, respectively). **d,** Plasma insulin levels at 0 and 15 min timepoints from OGTT experiment in males (n=12, 5 litters and n=10, 5 litters for control and STZ offspring, respectively). **e,** AUC from the OGTT experiment in females (n=6, 3 litters and n=7, 4 litters for control and STZ offspring, respectively). **f,** Plasma insulin levels at 0 and 15 min timepoints from OGTT experiment in females (n=6, 3 litters and n=7, 4 litters for control and STZ offspring, respectively). **c and d,** were analyzed using an unpaired student's t-test, **a, and b,** were analyzed using a repeated measures two-way ANOVA, and **d,** and **f,** were analyzed using a repeated measures two-way ANOVA with Bonferroni's post-hoc test. Data are represented as mean +/- SEM.

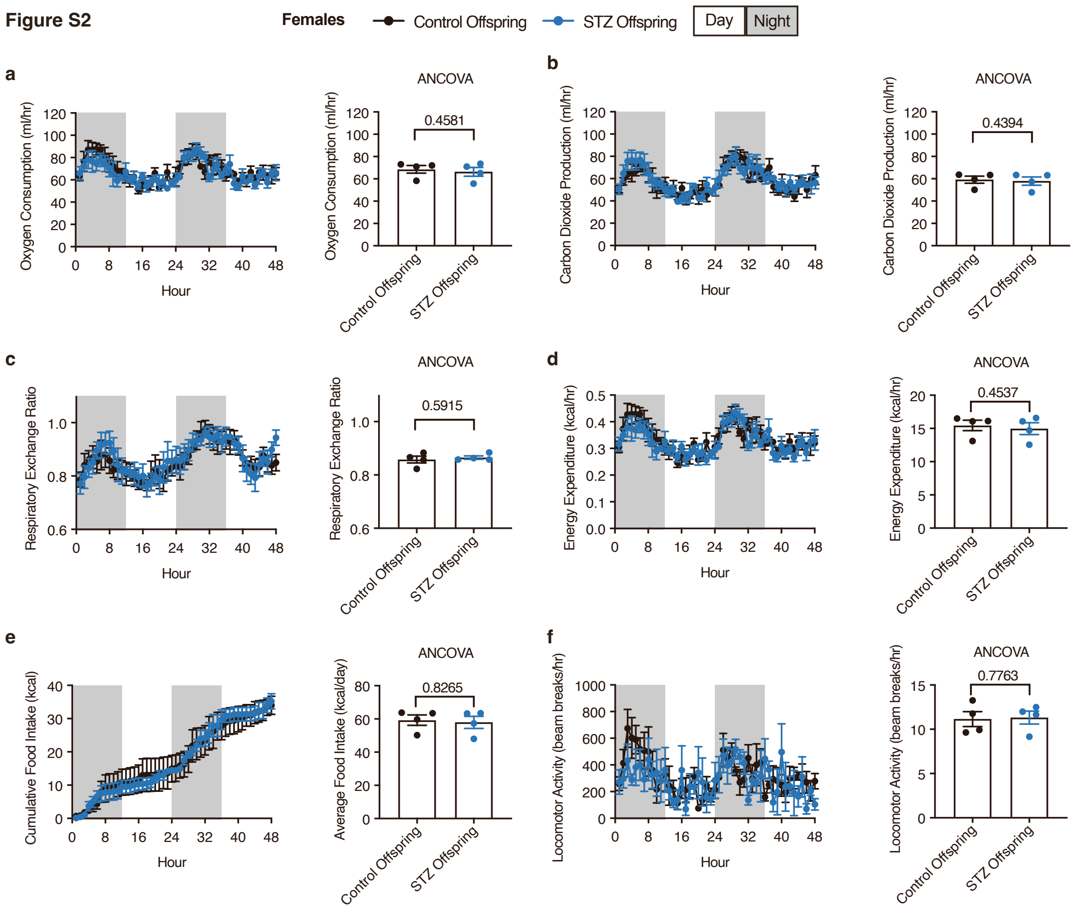

Figure S2. Indirect calorimetry of female control and STZ offspring. Related to Figure 2. a, Oxygen consumption rate in female control (n=4, n=3 litters) and STZ offspring (n=4, n=3 litters). **b,** Carbon dioxide production in female control and STZ offspring. **c,** Energy expenditure in female control and STZ offspring. **d,** Respiratory exchange ratio in female control and STZ offspring. **e,** Cumulative food intake in female control and STZ offspring. **f,** Locomotor activity in female control and STZ offspring. Data analyzed using an ANCOVA with total mass of animals as a covariate. Data are represented as mean +/- SEM.

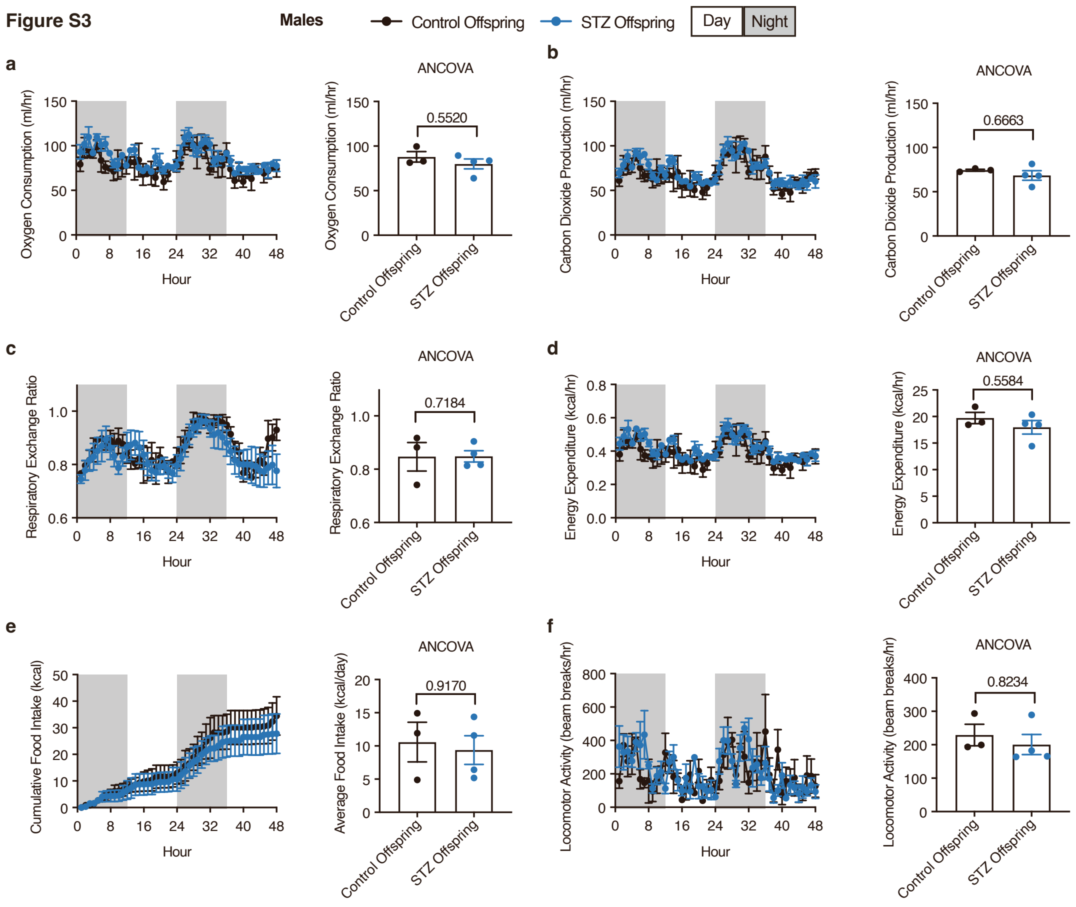

Figure S3. Indirect calorimetry of male control and STZ offspring. Related to Figure 2. a, Oxygen consumption rate in male control (n=3, n=3 litters) and STZ offspring (n=4, n=3 litters). **b,** Carbon dioxide production in male control and STZ offspring. **c,** Energy expenditure in male control and STZ offspring. **d,** Respiratory exchange ratio in male control and STZ offspring. **e,** Cumulative food intake in male control and STZ offspring. **f,** Locomotor activity in male control and STZ offspring. Data analyzed using an ANCOVA with total mass of animals as a covariate. Data are represented as mean +/- SEM.

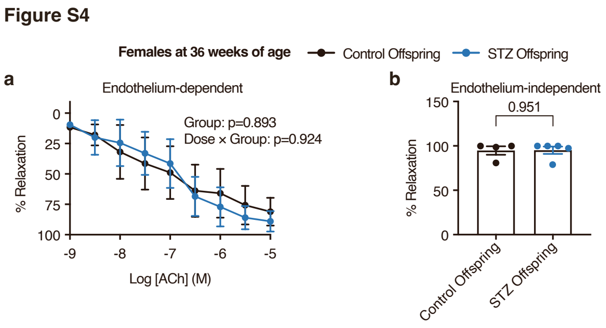

Figure S4. No differences in EDR between female control and STZ offspring at a more advanced age. Related to Figure 3. a, Endothelium-dependent relaxation (EDR) in mouse aortas from female offspring of female control (n=4, n=3 litters) and STZ mice (n=5, n=3 litters) evoked with acetylcholine (ACh) at 36 weeks of age. **b,** Maximum endothelium-independent relaxation (EIR) evoked with a single max dose of sodium

nitroprusside (10^{-5} M). Data in **a,** was analyzed using a repeated measures two-way ANOVA, data in **b,** was analyzed using an unpaired student's t-test. Data are represented as mean +/- SEM.

Figure S5

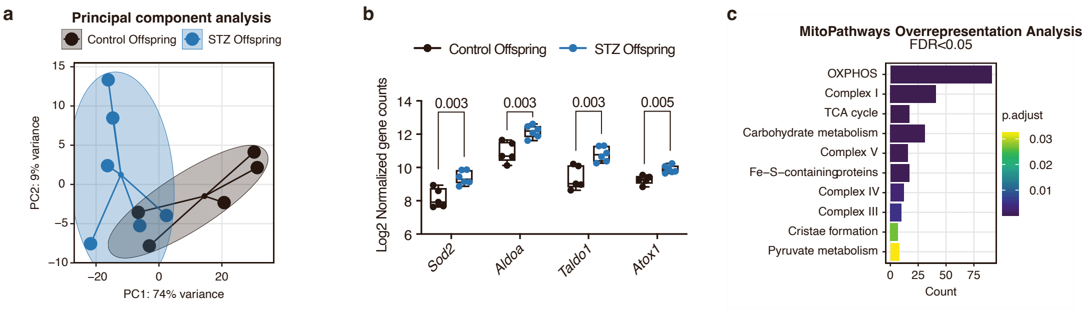

Figure S5. Aorta transcriptomic analysis. Related to Figure 3. a, Principal component analysis (PCA) plot of aortas from control and STZ offspring analyzed using RNA sequencing. **b,** Box plots depicting log2 normalized gene counts of differentially expressed genes (DEGs) of interest. **c,** Barplot depicting significantly enriched MitoPathways in upregulated DEGs using overrepresentation analysis. Data in **b,** was analyzed using a Wald test in DESeq2. Data are represented as boxplots.

Figure S6

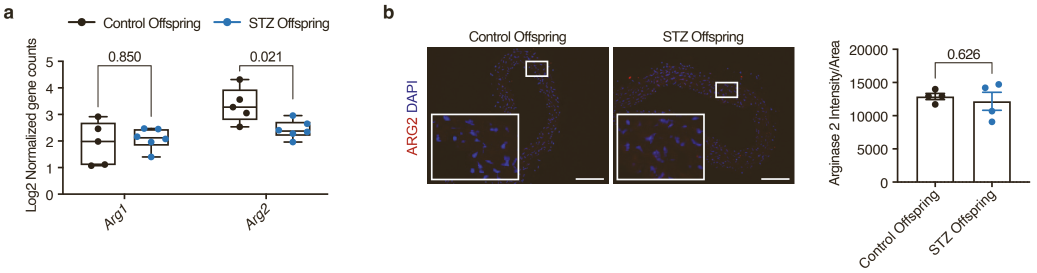

Figure S6. No differences found in *Arg1* gene expression and Arginase 2 protein levels between male control and STZ offspring. Related to Figure 3. a, Box plots depicting log2 normalized gene counts of *Arg1*and *Arg2* in male control and STZ offspring aortas. **b,** Representative images of staining of Arg2 (red) in aortic rings from male control and STZ offspring, scale bars denote 100 µm and quantification of Arg2 in tissue sections of aortas from male control (n=5, n=3 litters) and STZ (n=6, n=3 litters) offspring. Data in **a,** was analyzed using a Wald test in DESeq2, data in **b,** was analyzed using an unpaired student's t-test. Data are represented as mean +/- SEM in **b,** and boxplots in **a,**.

Figure S7

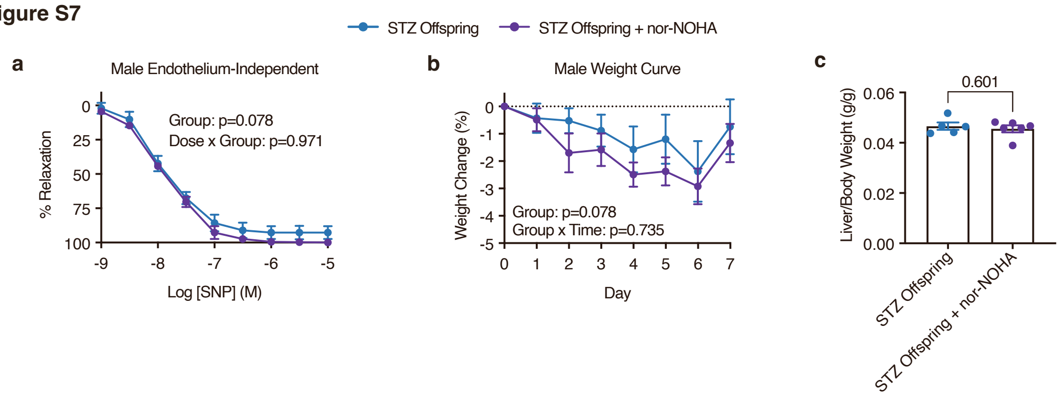

Figure S7. No differences in endothelium-independent relaxation (EIR), body weight and liver weight between male STZ offspring treated with either PBS or nor-NOHA. Related to Figure 3. a, EIR in male STZ offspring aortas treated with PBS (n=4, n=3 litters) or nor-NOHA (n=6, n=3 litters). **b,** Body weight change after start of injections in male STZ offspring treated with PBS (n=5, n=3 litters) or nor-NOHA (n=6, n=3 litters). **c,** Liver weight divided by body weight in male STZ offspring treated with PBS (n=5, n=3 litters) or nor-NOHA (n=6, n=3 litters). Data in **a,** and **b,** was analyzed using a repeated measures two-way ANOVA, data in **c,** was analyzed using an unpaired student's t-test. Data are represented as mean +/- SEM.

Table S1. Related to Table 1 and Figure 4. – Characteristics of study participants according to maternal and paternal type 1 diabetes status in Swedish data.

Basic characteristics	Maternal T1D during pregnancy		Paternal T1D during pregnancy	
	Unexposed	Exposed	Unexposed	Exposed
Men, n (%)	1,566,125 (51)	2,391 (50)	1,498,665 (51)	4,470 (51)
Year of birth, n (%)				
1982-1989	704,864 (23)	696 (15)	655,276 (23)	972 (11)
1990-1994	530,551 (17)	280 (6)	500,782 (17)	1,105 (13)
1995-1999	408,851 (13)	363 (8)	390,354 (13)	1,042 (12)
2000-2004	422,812 (14)	836 (18)	409,012 (14)	1,406 (16)
2005-2009	474,362 (16)	1,099 (23)	462,987 (16)	1,936 (22)
2010-2014	504,824 (17)	1,474 (31)	496,136 (17)	2,303 (26)
Gestational age in days, mean (SD)	279.0 (12)	269.4 (15)	278.9 (12)	279.1 (12)
Birth weight for gestational age, n (%)				
Small for gestational age	71,280 (2)	55 (1)	66,262 (2)	168 (2)
Normal	2,869,974 (94)	3310 (70)	2,740,308 (94)	8,288 (95)
Large for gestational age	92,747 (3)	1356 (29)	96,465 (3)	283 (3)
Unknown	12,223 (0)	27 (1)	11,512 (0)	25 (0)
Birth order, n (%)				
1	1,306,596 (43)	2,212 (47)	1,258,573 (43)	4,145 (47)

2	1,112,384 (37)	1,693 (36)	1,065,217 (37)	3,220 (37)
≥3	627,244 (21)	843 (18)	590,757 (20)	1399 (16)
Maternal age in years, mean (SD)	29.6 (5)	29.8 (5)	29.6 (5)	29.4 (5)
Maternal post-secondary education or above, n (%)	1,121,676 (37)	2,017 (43)	1,085,385 (38)	3,738 (43)
Paternal post-secondary education or above, n (%)	982,315 (33)	1,591 (34)	951,811 (33)	3,140 (36)
Maternal smoking, n (%)				
Never	2,403,943 (79)	3,906 (82)	2,307,321 (79)	7,261 (83)
1-9 cigarettes per day	290,184 (10)	357 (8)	274,583 (9)	689 (8)
≥10 cigarettes per day	150,793 (5)	155 (3)	141,780 (5)	326 (4)
Maternal BMI during pregnancy, mean (SD)	23.7 (4)	24.9 (4)	23.7 (4)	24.0 (4)
Caesarean section, n (%)	392,625 (13)	1,951 (41)	382,645 (13)	1,249 (14)
Maternal CVD, n (%)	479,514 (16)	1,111 (23)	469,707 (16)	1,147 (13)
Paternal CVD, n (%)	584,650 (19)	673 (14)	501,948 (17)	3042 (35)
Paternal lifetime diabetes, n (%)				
No	2,818,702 (93)	4,472 (94)	-	-
T1D	9,435 (0)	35 (1)	-	-
T2D	218,087 (7)	241 (5)	-	-
Maternal lifetime diabetes, n (%)				
No	-	-	2,818,728 (97)	8,553 (98)
T1D	-	-	5,570 (0)	41 (1)
T2D	-	-	90,249 (3)	170 (2)

T1D: type 1 diabetes; SD: standard deviation; BMI: body mass index; T2D: type 2 diabetes; CVD: cardiovascular disease.

Table S2. Related to Table 1 and Figure 4. – Characteristics of study participants according to maternal and paternal type 1 diabetes status in Danish data.

Basic characteristics	Maternal T1D during pregnancy		Paternal T1D during pregnancy	
	Unexposed	Exposed	Unexposed	Exposed
Men, n (%)	603,555 (51)	802 (53)	607,413 (51)	1,491 (51)
Year of birth, n (%)				
1997-1999	186,511 (16)	229 (15)	185,827 (16)	370 (13)
2000-2004	300,671 (26)	386 (26)	299,448 (25)	707 (24)
2005-2009	288,151 (24)	363 (24)	290,407 (25)	760 (26)
2010-2014	256,026 (22)	327 (22)	260,528 (22)	692 (24)
2015-2017	145,630 (12)	205 (14)	148,111 (13)	406 (14)
Gestational age in days, mean (SD)	279.03 (11.85)	263.04 (13.82)	278.92 (11.91)	279.03 (11.69)
Birth weight for gestational age, n (%)				
Small for gestational age	107,468 (9)	58 (4)	107,528 (9)	256 (9)

Normal	934,813 (79)	780 (52)	939,703 (78)	2,352 (80)
Large for gestational age	113,570 (10)	660 (44)	115,951 (10)	291 (10)
Unknown	21,138 (2)	12 (1)	21,139 (2)	36 (1)
Birth order, n (%)				
1	511,050 (43)	758 (50)	515,823 (44)	1,348 (46)
2	423,782 (36)	545 (36)	426,705 (36)	1,102 (38)
≥3	209,802 (18)	190 (13)	209,359 (18)	421 (14)
Unknown	32,355 (3)	17 (1)	32,434 (3)	64 (2)
Maternal age in years, mean (SD)	29.21 (4.85)	29.44 (4.64)	29.21 (4.84)	29.08 (4.51)
Maternal post-secondary education or above, n (%)	428,678 (36)	572 (38)	433,646 (37)	1,165 (40)
Paternal post-secondary education or above, n (%)	355,294 (30)	467 (31)	358,450 (30)	1,008 (34)
Maternal smoking, n (%)				
No	891,825 (76)	1,130 (75)	898,698 (76)	2,333 (79)
Yes	164,818 (14)	218 (14)	165,304 (14)	377 (13)
Unknown	120,346 (10)	162 (11)	120,319 (10)	225 (8)
Maternal BMI in kg/m^2 during pregnancy, mean (SD)	23.74 (5.95)	23.99 (6.14)	23.78 (6.02)	24.04 (8.05)
Caesarean section, n (%)	207,713 (18)	785 (52)	211,434 (18)	530 (18)
Maternal CVD, n (%)	90,358 (8)	355 (24)	91,702 (8)	228 (8)
Paternal CVD, n (%)	125,855 (11)	168 (11)	123,012 (10)	836 (28)
Paternal lifetime diabetes, n (%)				
No	1,127,634 (96)	1,461 (97)	-	-
T1D	3,043 (0)	5 (0)	-	-
Other than T1D	46,312 (4)	44 (3)	-	-
Maternal lifetime diabetes, n (%)				
No	-	-	1,140,017 (96)	2,824 (96)
T1D	-	-	1,619 (0)	5 (0)
T2D	-	-	42,685 (4)	106 (4)

T1D: type 1 diabetes; SD: standard deviation; BMI: body mass index; T2D: type 2 diabetes; CVD: cardiovascular disease.
